# Heterogeneity of Synovial Molecular Patterns in Patients with Arthritis

**DOI:** 10.1371/journal.pone.0122104

**Published:** 2015-04-30

**Authors:** Bernard R. Lauwerys, Daniel Hernández-Lobato, Pierre Gramme, Julie Ducreux, Adrien Dessy, Isabelle Focant, Jérôme Ambroise, Bertrand Bearzatto, Adrien Nzeusseu Toukap, Benoît J. Van den Eynde, Dirk Elewaut, Jean-Luc Gala, Patrick Durez, Frédéric A. Houssiau, Thibault Helleputte, Pierre Dupont

**Affiliations:** 1 Pôle de pathologies rhumatismales, Institut de Recherche Expérimentale et Clinique, Université catholique de Louvain and Department of Rheumatology, Cliniques Universitaires Saint-Luc, Brussels, Belgium; 2 Machine Learning Group, ICTEAM Institute, Université catholique de Louvain, Place Sainte-Barbe 2, B-1348, Louvain-la-Neuve, Belgium; 3 DNAlytics, Louvain-la-Neuve, Belgium; 4 Centre de Technologies Moléculaires Appliquées, Institut de Recherche Expérimentale et Clinique, Université catholique de Louvain, Brussels, Belgium; 5 Institut de Duve, Université catholique de Louvain, Brussels, Belgium; 6 Rheumatology Department, Ghent University Hospital, Ghent, Belgium; University of Texas Health Science Center at Houston, UNITED STATES

## Abstract

**Objectives:**

Early diagnosis of rheumatoid arthritis (RA) is an unmet medical need in the field of rheumatology. Previously, we performed high-density transcriptomic studies on synovial biopsies from patients with arthritis, and found that synovial gene expression profiles were significantly different according to the underlying disorder. Here, we wanted to further explore the consistency of the gene expression signals in synovial biopsies of patients with arthritis, using low-density platforms.

**Methods:**

Low-density assays (cDNA microarray and microfluidics qPCR) were designed, based on the results of the high-density microarray data. Knee synovial biopsies were obtained from patients with RA, spondyloarthropathies (SA) or osteoarthritis (OA) (n = 39), and also from patients with initial undifferentiated arthritis (UA) (n = 49).

**Results:**

According to high-density microarray data, several molecular pathways are differentially expressed in patients with RA, SA and OA: T and B cell activation, chromatin remodelling, RAS GTPase activation and extracellular matrix regulation. Strikingly, disease activity (DAS28-CRP) has a significant influence on gene expression patterns in RA samples. Using the low-density assays, samples from patients with OA are easily discriminated from RA and SA samples. However, overlapping molecular patterns are found, in particular between RA and SA biopsies. Therefore, prediction of the clinical diagnosis based on gene expression data results in a diagnostic accuracy of 56.8%, which is increased up to 98.6% by the addition of specific clinical symptoms in the prediction algorithm. Similar observations are made in initial UA samples, in which overlapping molecular patterns also impact the accuracy of the diagnostic algorithm. When clinical symptoms are added, the diagnostic accuracy is strongly improved.

**Conclusions:**

Gene expression signatures are overall different in patients with OA, RA and SA, but overlapping molecular signatures are found in patients with these conditions. Therefore, an accurate diagnosis in patients with UA requires a combination of gene expression and clinical data.

## Introduction

Rheumatoid arthritis (RA) is an inflammatory joint disorder that results in progressive joint damage when insufficiently treated. In order to prevent joint destruction and functional disability in RA, early diagnosis and initiation of appropriate treatment with Disease-Modifying Anti-Rheumatic Drugs (DMARDs) is needed [1–5]. However, in daily clinical practice, patients may initially display symptoms of arthritis that do not fulfill the classification criteria for a definite diagnosis of RA, or any other joint disease, a situation called “Undifferentiated Arthritis” (UA). Out of the patients with UA, 30 to 50% usually develop RA, and early identification of these remains a challenge [6].

At the present time, although several risk factors associated with the development of RA have been identified [6–9], a model that reliably predicts the probability of evolution of UA into RA in individual patients is lacking. In order to better identify early RA patients, an American College of Rheumatology (ACR)/European League Against Rheumatism (EULAR) collaboration recently developed new RA classification criteria [10]. Although these criteria are more sensitive, the risk of misdiagnosis is an important issue to consider, especially in very early disease [11, 12]. In this context, the present study explores the feasibility of a molecular diagnosis of arthritis, based on the identification of disease-specific transcriptomic profiles in synovial biopsies from UA patients, using low-density cDNA arrays.

We performed global analyses of gene expression in synovial biopsies from patients with RA, Systemic Lupus Erythematosus (SLE), Osteoarthritis (OA), psoriatic arthritis (SA) and microcrystalline arthritis (MIC), using high-density oligonucleotide spotted microarrays [13]. We found that the gene expression profiles were strikingly different according to the underlying condition, and we used these data in order to identify disease-specific molecular signatures. Out of the disease-specific probes, 100 were spotted on two different low-density gene expression platforms (cDNA array and microfluidics qPCR) in order to evaluate their diagnostic accuracy in an independent set of patients. Our results confirm overall differences in gene expression in patients with RA, SA and OA. However, transcriptomic overlaps among the different clinical entities result in a diagnostic accuracy that does not exceed 57% when using transcriptomic data alone. In order to reach a high diagnostic performance, addition of clinical data into the diagnostic algorithm is needed.

## Materials and Methods

### Synovial biopsies and RNA extraction

Synovial biopsies were obtained by needle arthroscopy of the affected knee. For each procedure, 6 to 8 biopsy fragments were stored in RNA later and frozen at −80°. Synovial biopsy samples were homogenized using an Ultra-Turrax rotor (Ika, Staufen, Germany) and total RNA was extracted from synovial biopsy tissue using the Nucleospin RNA II extraction kit (Macherey-Nagel, Düren, Germany), including DNase treatment of the samples. RNA quantity was assessed by optical density measurements, using a Nanodrop ND-1000 spectrophotometer (Thermo Fisher Scientific Inc., Wilmington, DE). RNA quality was evaluated by capillary electrophoresis using an Agilent 2100 Bioanalyzer and RNA Nano Chips (Agilent Technologies Inc., Santa Clara, CA). All samples had a RNA integrity number (RIN) higher than 6.

### High-density microarray studies

GeneChip Human Genome U133 Plus 2.0 Arrays (Affymetrix UK Ltd., High Wycombe, UK) were hybridized in monoplicates with 10 μg biotinylated cRNA, synthesized as previously described [13], using synovial biopsy samples obtained in untreated patients with RA (n = 7), OA (n = 5), SLE (n = 4), SA (based on the presence of peripheral arthritis and skin psoriasis; n = 4) and MIC (based on the identification of urate or calcium pyrophosphate crystals in the synovial fluid, n = 5). The gene expression data observed in the patients with RA, OA and SLE were previously reported in [13]. The slides were stained using the EukGE-WS2v5 Fluidics protocol on the GeneChip Fluidics Station (Affymetrix UK Ltd.), and scanned on a GeneChip Scanner 3000, resulting in the generation of .CEL files used in the analyses described below. All the data used in this experiment were deposited in the Gene Expression Omnibus (GEO) of the National Center for Biotechnology Information, and are accessible through GEO series accession number [GEO: GSE36700].

In addition, 32 GeneChip Human Genome U133 Plus 2.0 data, obtained by our group in synovial biopsies from untreated early (< 1 year disease duration, n = 20) [14], and from refractory (resistant to methotrexate and TNF blockade, n = 12) [15] RA patients were retrieved from [GEO: GSE45867] and [GEO: GSE24742].

### Low-density microarray studies

93 biopsies from 88 additional patients with knee synovitis were included in this part of the study. All were untreated at the time of the biopsy (at the exception of non-steroidal anti-inflammatory drugs). Out of them, 39 had a definite diagnosis: RA (n = 10), according to the 1987 American College of Rheumatology (ACR) classification for RA [16]; SA (n = 4), based on the presence of psoriasis or typical x-ray evidence of peripheral or sacro-iliacal joint involvement, and OA (n = 25), based on x-ray evidence. There were no biopsies available from patients with MIC and SLE.

The 49 remaining patients had UA. A clinical diagnosis was obtained for all of them, after a median follow-up of 9 months: RA (n = 21), OA (n = 19) and SA (n = 9). 5 of these UA patients, who would later develop RA, had a repeat biopsy after 3 months. They had received placebo treatment in the context of a randomized trial, and were therefore still untreated at the time of the second biopsy. Therefore, 54 distinct UA samples were used, 26 of which were harvested in patients with a later diagnosis of RA.

Baseline characteristics of the patients are described in Tables 1 and 2. The study was approved by the ethics committee of the Université catholique de Louvain, and written informed consent was obtained from all patients.

**Table 1:** Baseline characteristics of the patients with a known diagnosis at the time of the needle-arthroscopy procedure.

Characteristics	RA (n = 10)	SA (n = 4)	OA (n = 25)
Age in years, mean ± SD	52.5 ±24.4	45.5 ±18.5	65.2 ±8.3
Female gender n / %	7 / 70.0%	1 / 25%	19 / 76.0%
ACR 1987 RA criteria positivity n / %	10 / 100%	0 / 0%	0 / 0%
ACR/EULAR criteria 2010 n / %	10 / 100%	0 / 0%	0 / 0%
Morning stiffness > 1hour n / %	9 / 90%	1 / 25%	2 / 8.0%
Swollen joint count (28 assessed), mean ± SD	8.4 ±3.5	1.75 ±0.5	1.6 ±1.4
Arthritis of hands n / %	10 / 100%	0 / 0%	1 / 4.0%
Symmetrical involvement of joints n / %	10 / 100%	2 / 50%	9 / 36.0%
Psoriasis n / %	0 / 0%	4 / 100%	0 / 0%
ACPA positivity n (%)	8 / 80%	0 / 0%	0 / 0%
Rheumatoid factor positivity n / %	9 / 90%	0 / 0%	3 / 12.0%
X-ray erosions n / %	6 / 60%	0 / 0%	0 / 0%
CRP > normal value n / %	10 / 100%	1 / 25%	1 / 4.0%

**Table 2:** Baseline characteristics of the patients with UA, categorized according to the development of RA after a median follow-up of 9 months (range – months).

Characteristics	No progression to RA (n = 28)	Progression to RA (n = 26)
Age in years, mean +/- SD	48.7 ± 14.1	43.7 ± 9.2
Female gender n / %	16 / 57.1%	19 / 73.1%
Morning stiffness > 1hour n / %	6 / 21.4%	12 / 46.1%
Swollen joint count (28 assessed) +/- SD	2.4 ± 2.5	3.0 ± 2.7
Arthritis of hands n / %	4 / 14.3%	19 / 73.1%
Symmetric involvement of joints n / %	12 / 42.9%	8 / 30.8%
Psoriasis n / %	3 / 10.7%	0 / 0%
ACPA positivity n / %	0 / 0%	20 / 76.9%
Rheumatoid factor positivity n / %	2 / 7.1%	19 / 73.1%
ACR/EULAR 2010 criteria positivity n / %	1 / 3.6%	11 / 42.3%
X-ray erosions n / %	0 / 0%	3 / 11.5%
CRP > normal value n / %	5 / 17.9%	13/ 50.0%

Total RNA (1 μg) was amplified using the Ambion MessageAmp II aRNA Amplification Kit (Invitrogen Life Sciences, Gent, Belgium), and aRNA was used for the synthesis of biotinylated cDNA, using the DualChip cDNA synthesis protocol (Eppendorf Array Technologies, Namur, Belgium). Briefly, the aRNA template was spiked with a mix of *in vitro* transcribed polyadenylated RNA as internal standard (Silverquant internal standards, Eppendrof Array Technologies, Namur, Belgium), and the synthesis of biotinylated cDNA was carried out using oligo(dT) primers and Superscript II reverse transcriptase (Invitrogen) in the presence of a dNTP mix, together with biotin-11-dCTP and biotin-11-dATP. At the end of the reaction, RNAse H was added for 20 minutes, and the reaction was terminated by heating the mix for 3 minutes at 95°.

The biotinylated cDNA samples were spiked with biotinylated hybridization controls (Biotin-HybControl, Eppendorf Array Technologies, Namur, Belgium), and hybridized overnight in monoplicates on customized DualChip low-density arrays, spotted with 200 bp-long cDNA probes targeting 100 diagnostic genes, together with 68 probes targeting house keeping genes (n = 13), internal standards (n = 6) and hybridization controls (n = 49) (all probes are spotted in triplicates on each slide). The slides were washed, and incubated in the presence of a anti-biotin antibody with nanogold particle conjugates. Finally, the slides were stained by the addition of a Silverquant silver reagent that precipitates in the presence of nanogold particles. Colorimetric detection of the spot intensities was performed on a Silverquant Scanner (Eppendorf Array Technologies). Raw gene intensity data are displayed in S1 Table.

### Low density qPCR studies

31 biopsies from from patients (n = 29) with initial UA with knee synovitis were included in this part of the study. A repeat biopsy at 3 months was collected in 2 patients who had received placebo treatment in the context of a randomized trial. 30 of these samples were also hybridized on the low-density microarrays. All patients were untreated at the time of the biopsy. A clinical diagnosis was obtained in all of them after a median follow-up of 9 months: RA (n = 15), OA (n = 11), and SA (n = 5).

Total RNA (500 ng) was reverse-transcribed into cDNA, using the high-capacity reverse transcription kit (Life Technologies, Merelbeke, Belgium). A customized Taqman low-density array (Life Technologies) was designed in order to assess the expression of 95 signature and one house-keeping (18s) genes, using pre-designed Life Technologies Taqman assays. 16 ml cDNA was mixed with 84 ml H2O and 100 ml 2x Taqman universal master mix (Life Technologies). Each sample was loaded into two ports of the card, and run on an ABI 7900HT system for 2 min at 50°C, 10 min at 95°C, followed by 40 cycles for 15s at 97°C and 1 min at 60°C. One of the assays (MYEOV2) failed to generate any signal, and was excluded from the analyses. Raw qPCR data are displayed in S2 Table.

### Analyses of the high-density microarray data

The initial high-density array data consist of .CEL files (HGU133 Plus 2.0 slides) from 25 untreated patients diagnosed with RA (n = 7), OA (n = 5), SLE (n = 4), SA (n = 4), MIC (n = 5). Normalized gene expression data were obtained from the .CEL files using the RMA pre-processing method [17]. The data were randomly split 200 times into a training set and a test set containing respectively 90% and 10% of the samples. Then, for each partition of the data, two filtering approaches were employed to reduce the list of potential genes available for prediction: (i) A quantile filtering approach was used to remove 75% of the genes with the smallest variability across samples, where variability was measured in terms of the distance between the 5-th and the 95-th percentile; (ii) ANOVA filtering was employed next to remove those genes whose associated p-value was above 5%.

For each train and test partition of the data, and for progressively decreasing values (ranging from 1000 to 10) of the signature size (k), individual genes were ranked according to their power to discriminate each potential diagnosis, *i*.*e*. RA, SLE, OA, SA or MIC, from all the others. The statistic of a standard Student’s t-test was used for this purpose. For each potential diagnosis we selected the top k / 5 ranked genes, which were put together to obtain a signature of size k. Then, we evaluated the diagnostic performance associated to the signature on the test set and we reported average results.

The predictor employed in these analyses was a nearest neighbors classifier which uses the Pearson correlation distance. The diagnostic performance of the nearest neighbors classifier was measured in terms of the balanced classification rate (BCR). The BCR is defined as the arithmetic average of the accuracies within the samples corresponding to each potential diagnosis.

The variability in the expression of the signature genes was further evaluated in a second set of high-density array data, consisting of 32 .CEL files (HGU133 Plus 2.0 slides) originated from an independent set of RA patients.

### Analyses of the low-density microarray data

The low-density array data consist of 93 text files from 88 patients included in this part of the study. Raw data were pre-processed in four successive steps. In a first step, a standard background correction method was applied by subtracting the corresponding local background from each spot’s foreground intensity. In a second step, the generalized log (glog) transformation described in [18] was carried out in order to stabilize the variance on the whole range of signal intensity. Its parameters were tuned by maximizing likelihood, using the R package LMGene. In a third step, a scale normalization based on internal standards and house-keeping genes was applied. In particular, for each replicate z_ijk_, with k = 1, 2, 3, corresponding to patient i and internal standard or house keeping gene j, it was assumed that

$${\text{z}}_{\text{ijk}}=\mu +{\text{a}}_{\text{i}}+{\text{b}}_{\text{j}}+\sum_{\text{ijk}}$$

where μ is a constant bias term, a_i_ is a patient-specific parameter, b_j_ is a gene-specific parameter, ∑_ijk_ is standard residual Gaussian noise and, without loss of generality, a_1_ = b_1_ = 0. Parameters a_i_ and b_j_ were tuned by minimizing the sum of squared residuals. The fitting of the model was repeated after removing replicates whose residuals were outliers, in an iterative process until no such outliers were found. This gave coefficients a_i_ for each sample which were then subtracted from the data of the corresponding patient. Finally, triplicate spot intensities for each diagnostic gene were summarized in a single median value.

The diagnostic predictions were performed by randomly splitting the low-density array data 200 times into a training set and a test set containing respectively 90% and 10% of the samples. Using these different partitions of the data, we evaluated the diagnostic performance of a nearest neighbors classifier with 1, 3 or 5 nearest neighbors. The performance metric employed was again the balanced classification rate (BCR). The 3-class BCR was computed (RA versus OA versus SA), as well as the 2-class BCR (RA versus not RA). The 2-class BCR of the ACR-EULAR 2010 criterion for RA is also reported as a comparator [10].

In a second analysis, we used clinical symptoms in order to improve the diagnostic performance of the nearest neighbors classifier. For this purpose, we modified the distance function employed in such classifier to combine both types of data: *i*.*e*. the intensity values corresponding to the diagnostic genes and some extra clinical attributes. Consider two arbitrary data samples characterized by the vectors *x*_1_ and *x*_2_. The distance in the nearest neighbors classifier which combines both types of data was set to:

$$dist\left(x_{1},x_{2}\right)=\rho .dist_{E}\left(\phi \left(x_{1}\right),\phi \left(x_{2}\right)\right)+\left(1-\rho \right).dist_{P}\left(\omega \left(x_{1}\right),\omega \left(x_{2}\right)\right)$$

where *dist*_*E*_ (.,.) is the Euclidean distance, *dist*_*P*_ (.,.) is the Pearson correlation distance, *φ*(.) is a function that returns the clinical attributes associated to the given sample, *ω*(.) is a function that returns the intensity values of the diagnostic genes associated to the given sample and *ρ* ∈ [0, 1] is a parameter that determines the combination of the data. If *ρ* = 1, the clinical attributes are exclusively used for prediction. If *ρ* = 0, the low-density array data alone are used instead. The influence of the *ρ* parameter was measured by computing the BCR on each resampled test set for 21 values of *ρ* evenly distributed in the interval [0, 1].

The whole resampling procedure, with varying parameters, was applied on several subsets of low-density data. The number of nearest neighbors and combination parameter were tuned on the 39 samples from patients with a definite diagnosis. The model was then run after addition of an independent set of 54 samples obtained in patients with UA at baseline.

### Analysis of the qPCR data

The same resampling procedure was also applied using qPCR data of 31 samples from patients with UA. Similarly, we evaluated the combined distance and the influence of the combination parameter on 2- and 3-class BCR.

## Results

### High density array data

**Fig 1:**
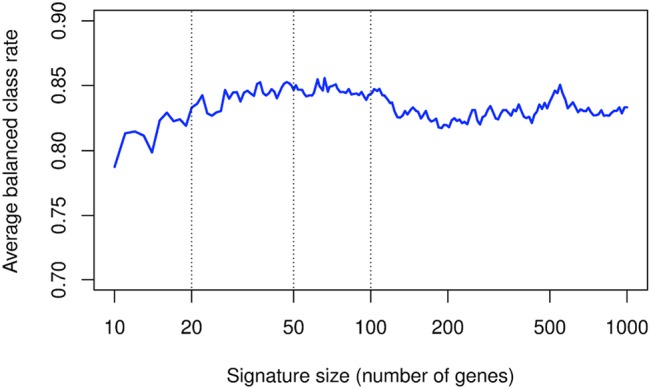
Balanced classification rate (BCR) of a nearest neighbor classifier as a function of the signature size (number of genes) used for prediction. Lists of genes of progressively decreasing sizes were determined based on high-density transcriptomic data, and used in order to predict diagnosis in 25 patients with RA, SLE, OA, SA and MIC. BCR is plotted in function of the signature size. Lists of genes containing between 20 and 100 probe sets provide performances that range between 83% and 85%.

**Fig 2:**
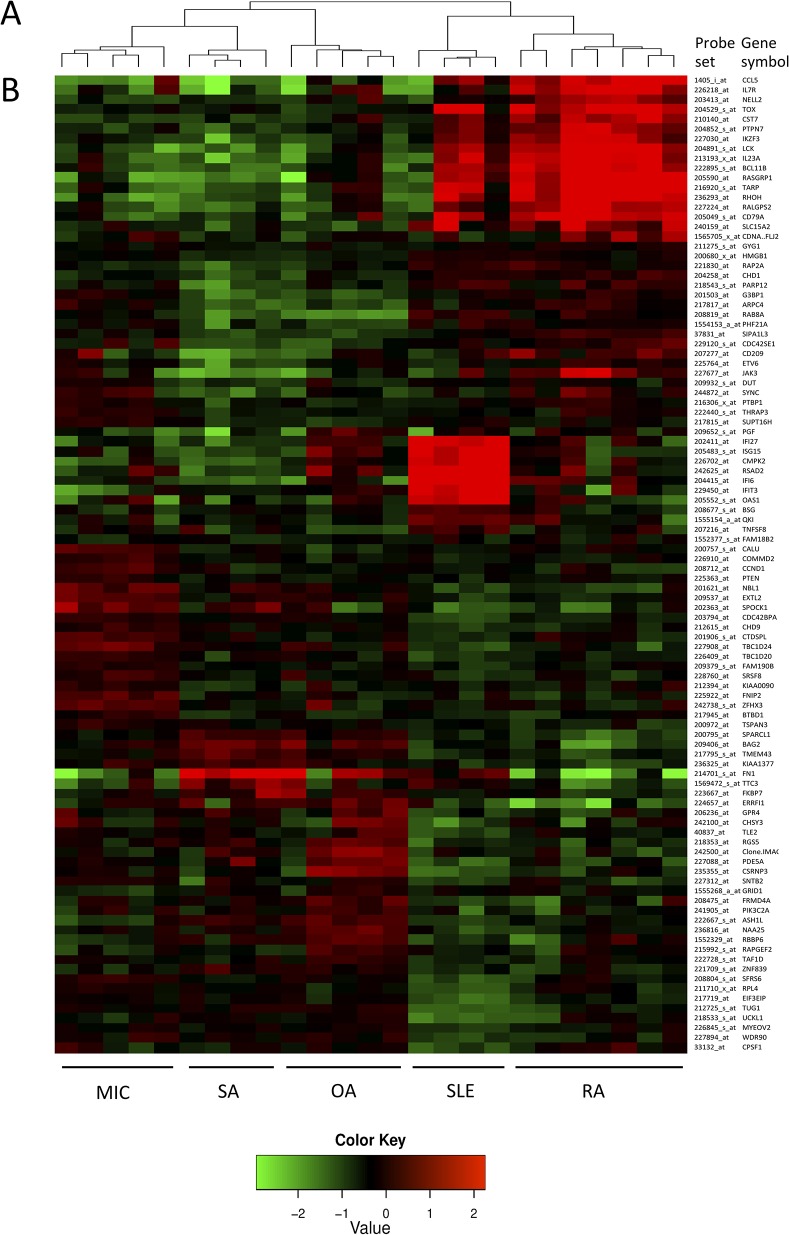
High-density gene expression data used for the design of the low-density platform. Analyses performed on high density transcriptomic data resulted in the selection of 100 probe sets differentiating patients with RA, SLE, OA, MIC and SA. The probes and gene symbols are also listed in S3 Table. (A) Hierarchical clustering algorithms using the high density gene expression values of these genes (and based on the Pearson correlation distance) distribute the samples into “inflammatory” (RA and SLE) and “high extra-cellular matrix turn-over” (OA, SA and MIC) clusters. They also identify diagnostic subdivisions. (B) The high density gene expression values of these 100 genes are displayed according to the clinical diagnosis of the samples.

In a first set of experiments, we used high-density transcriptomic data (HGU133 Plus 2.0 arrays) obtained in synovial biopsies from untreated patients with RA (n = 7), OA (n = 5), SLE (n = 4), SA (n = 4) and MIC (n = 5). After filtering the pooled data, we found by ANOVA that about 4,500 genes are differentially expressed among the 5 groups of patients. The cross-validation process was carried out in order to determine the minimal number of genes necessary for a right categorization. Fig 1 shows the BCR of a nearest neighbor classifier as a function of the size of the signature considered for prediction, using the high-density transcriptomic data. The figure shows that optimal diagnostic accuracies are obtained with signatures containing a number of genes that ranges between 20 and 100. Of note, the 100 most discriminant probes belong to pathways known to be relevant in the pathogenesis of several forms of arthritis: T and B cell activation (BCL11B, CD79A, IL7R, LCK, RHOH, IL23A, JAK3, CCL5), chromatin remodeling (PHF21A, ASH1L, CHD1, CHD9, HMGB1, SYNC, SUP16H, TOX), RAS GTPase activator activity (ERRFI1, RALGPS2, TBC1D24, TBC1D20), extracellular matrix (SPARCL1, FN1, SPOCK1), type I interferon-induced genes (IFI27, ISG15, RSAD2, IFI6, IFIT3, OAS1). The majority of the genes belonging to T and B cell activation / chromatin remodeling pathways are over-expressed in RA and SLE synovial biopsies, while type I interferon-induced genes are specifically over-expressed in SLE samples. By contrast, the genes over-expressed in RAS GTPase activator / extracellular matrix pathways are rather over-expressed in OA samples (Fig 2).

**Fig 3:**
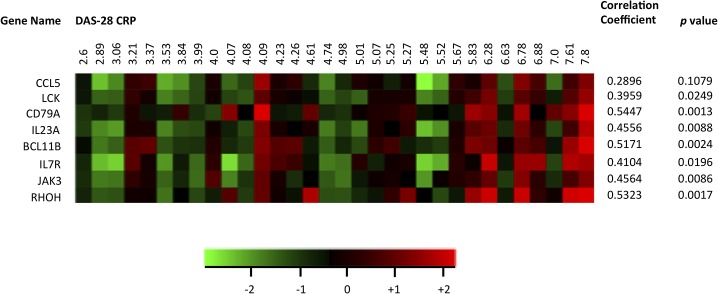
Effect of disease activity on gene expression in RA samples. Mean centered log2-transformed expression levels of selected T cell activation-associated transcripts were extracted from HGU133 Plus2.0 GeneChip array data sets of 32 patients with RA. DAS28-CRP scores were retrieved from the medical files of the patients, and the samples are sorted by ascending DAS28-CRP. Correlation coefficients (Pearson *r*) between gene expression and DAS28-CRP are displayed for each transcript.

Given the striking over-expression of specific gene subsets in these RA samples, we assessed their levels of expression in additional high-density transcriptomic data sets obtained in synovial biopsies from 32 other RA patients with active disease, prior to inclusion in therapeutic protocols. As shown in Fig 3, we could observe a significant heterogeneity in the expression of the selected T and B cell activation-associated genes among these samples. Interestingly, expression levels of the majority of these probe sets correlated significantly with disease activity (DAS28-CRP), thereby explaining part of the observed heterogeneity.

### Low density cDNA arrays

In order to further compare and explore the expression of the selected 100 genes in larger number of samples, we designed two low-density platforms (low density cDNA array and microfluidics qPCR). Expression profiling using the low-density array was first performed in 39 synovial samples obtained from untreated patients with a known diagnosis. These samples were different from these used in the high-density studies. Given the absence of biopsy samples from patients with MIC and SLE in these samples, we restricted the possible diagnostic choices to OA, RA and SA. Using the computation algorithm described in the Materials and Methods section (nearest neighbors classifier), the diagnostic performance of the array, measured in terms of the 3-class BCR, was 56.8%. Detailed performance measures (Table 3) show that a majority of prediction errors are confusions between RA and SA. All OA samples were correctly predicted, and 85.5% of the samples predicted as OA were indeed OA.

**Table 3:** Diagnostic performances of a 5 nearest neighbors classifier on clinical, trancriptomic (low-density arrays or qPCR), and combined data originated from synovial biopsy samples obtained in patients with a known diagnosis or UA.

Model	3 class BCR	2 class BCR	Sensitivity			Predictive positive value		
			RA	OA	SA	RA	OA	SA
**Known diagnosis – low density arrays (n = 39)**								
Clinical	94.1 ± 8.0	91.7 ± 12.0	86.5 ± 23.0	95.8 ± 7.4	100.0 ± 0.0	92.0 ± 14.0	96.5 ± 6.0	100.0 ± 0.0
Transcriptomic	56.8 ± 10.2	77.1 ± 15.2	70.5 ± 30.7	100.0 ± 0.0	0.0 ± 0.0	52.9 ± 21.3	85.5 ± 10.5	NA
Combined	**98.6 ± 2.5**	**98.4 ± 2.8**	**100.0 ± 0.0**	**95.8 ± 7.4**	**100.0 ± 0.0**	**93.8 ± 11.1**	**100.0 ± 0.0**	**100.0 ± 0.0**
**UA – low-density arrays (n = 54)**								
Clinical	64.0 ± 13.2	71.7 ± 9.7	62.0 ± 18.2	72.0 ± 21.1	58.0 ± 32.7	81.6 ± 13.7	51.8 ± 16.6	100.0 ± 0.0
Transcriptomic	47.1 ± 10.1	60.6 ± 11.9	59.0 ± 16.9	74.2 ± 19.3	8.0 ± 8.0	63.2 ± 15.0	54.6 ± 14.9	43.1 ± 36.9
Combined	**69.9 ± 13.4**	**80.1 ± 10.3**	**77.2 ± 15.8**	**74.5 ± 20.1**	**58.0 ± 32.7**	**84.8 ± 12.2**	**62.2 ± 17.6**	**100.0 ± 0.0**
ACR/EULAR 2010 criteria		67.9 ± 9.7	39.8 ± 18.2			92.1 ± 14.2		
**UA – qPCR (n = 31)**								
Clinical	74.3 ± 16.5	79.2 ± 16.1	74.0 ± 29.0	69.0 ± 30.6	80.0 ± 26.5	76.5 ± 21.7	65.7 ± 25.1	100.0 ± 0.0
Transcriptomic	38.7 ± 15.7	47.9 ± 19.7	48.5 ± 33.1	60.5 ± 32.4	7.0 ± 7.0	30.0 ± 21.8	47.8 ± 23.8	58.7 ± 39.6
Combined	**82.3 ± 15.4**	**90.1 ± 11.3**	**93.5 ± 16.3**	**73.5 ± 29.2**	**80.0 ± 26.5**	**84.9 ± 17.0**	**79.5 ± 22.7**	**100.0 ± 0.0**
ACR/EULAR 2010 criteria		69.9 ± 18.5	51.0 ± 33.1			72.3 ± 27.2		

The reported indicators (mean ± SD) were evaluated by multiple resampling, as described in the Materials and Methods section. The ACR/EULAR 2010 criteria for the diagnosis of RA are indicated as a comparator.

**Fig 4:**
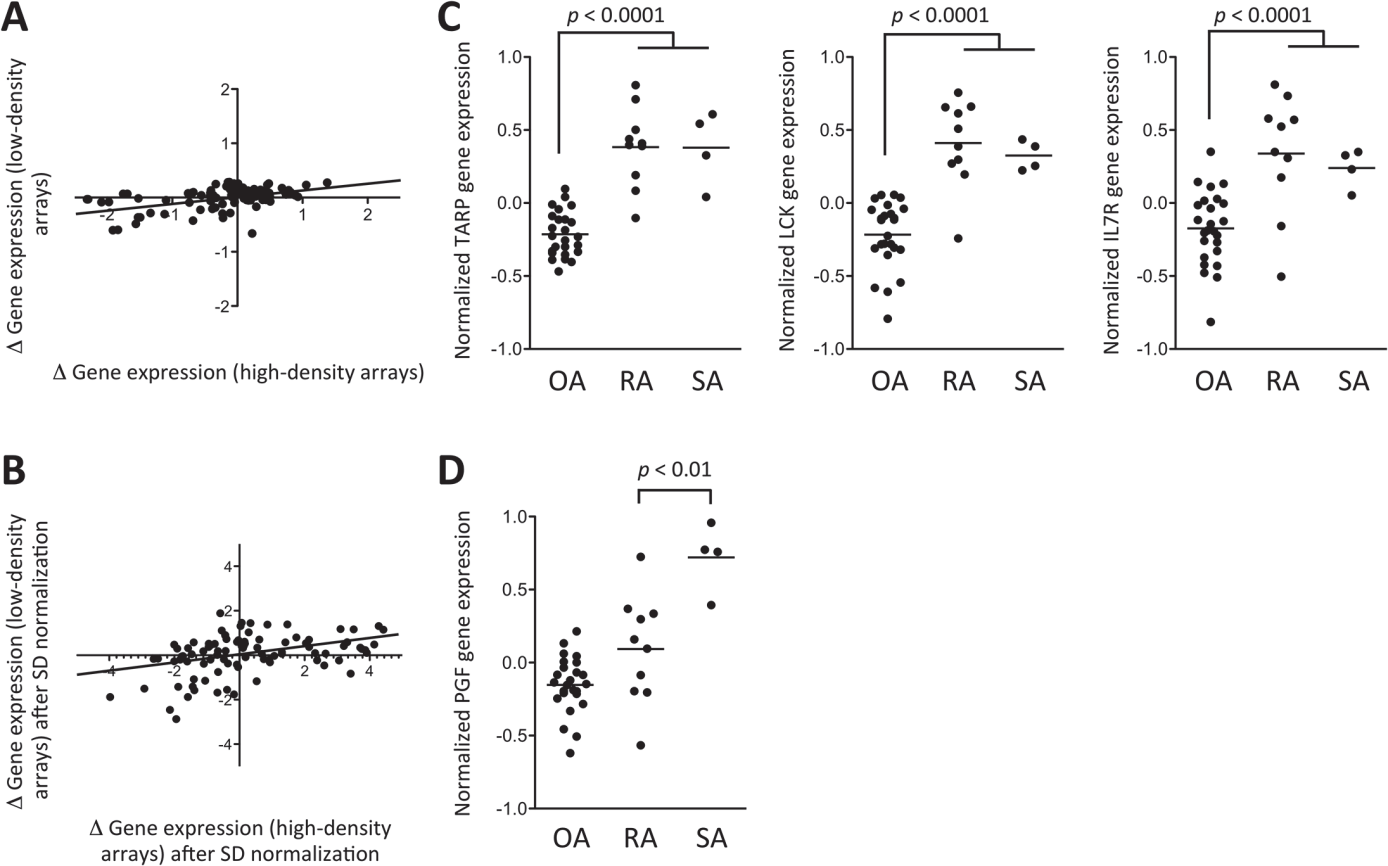
Comparison of gene expression differences between samples from patients with OA, RA and SA, using high-density versus low-density microarrays. Independent sets of samples were hybridized on high-density (HGU133 Plus 2.0 GeneChip) and low-density (DualChip) microarrays. (A) Differences in mean (log2-transformed) gene expression values between OA and (RA+SA) samples are displayed for the samples hybridized on high-density (x axis) versus low-density (y axis) arrays. (B) The same data from OA and (RA+SA) samples are displayed after normalization of each mean (log2-transformed) gene expression value by its standard deviation. (C) Normalized TCR gamma alternate reading frame protein (TARP), lymphocyte-specific protein tyrosine kinase (LCK) and Interleukin-7 Receptor (IL7R) gene expression data in OA versus RA and SA samples observed using low-density arrays. (D) Normalized Placental Growth Factor (PGF) gene expression data in OA versus RA and SA samples observed using low-density arrays. Mean values are represented by a horizontal bar. *p* values are calculated using Student’s t tests.

In order to explain the relative low performance of the computation algorithm, we compared the gene expression data obtained using the low-density arrays, to the original set of data from the high-density arrays (i.e. different platforms and different samples). The differences in gene expression in OA versus RA + SA samples displayed a good correlation (Pearson *r* = 0.47, *p* < 0.0001) between the two sets of data (Fig 4A). However, a decrease of the fold-change magnitude was observed in the low-density array data, as compared to the high-density data, and this compression of the fold change magnitudes in the low-density array data remained after normalization of the mean gene expression values by their standard deviations (Fig 4B). Similarly, there was a good correlation in the differences observed in RA versus SA samples (Pearson *r* = 0.36, *p* < 0.0005) between both platforms. Again, we observed a fold-change compression in the low-density arrays, and only a few genes displayed significant differences in gene expression between the two groups of patients in this set of data (data not shown). In addition, although we confirmed that OA samples were characterized by an overall over-expression of genes involved in RAS-GTPase activator activity and extracellular matrix regulation, while RA samples over-expressed genes involved in T and B cell activation, we observed individual overlaps in gene expression profiles that also contributed to the confusion of the computation algorithm (S1 Fig).

**Fig 5:**
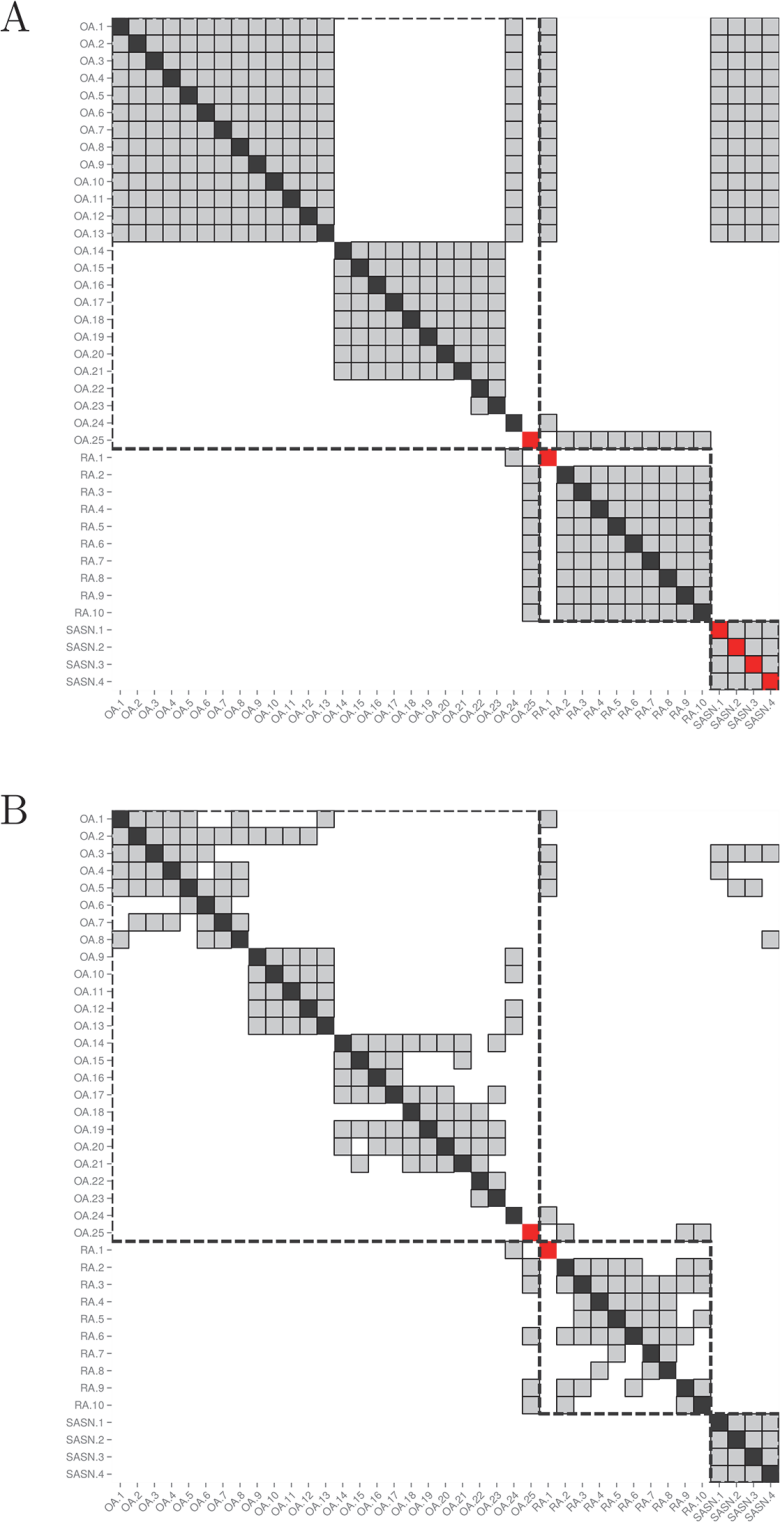
Impact of low-density microarray data on the determination of the nearest neighbors. Both matrices show the nearest neighbors of each biopsy sample from the cohort of patients with a known diagnosis (n = 39). Each sample is represented by a column, and its nearest neighbors are greyed out in that column. The cell on the diagonal is red if the sample is misclassified and black otherwise. Samples from patients with the same diagnosis are surrounded by a dashed square. (A) Nearest neighbors are determined using clinical data only (*ρ* = 1). More than 5 nearest neighbors are displayed for each sample due to the presence of ties. (B) Nearest neighbors are determined using a combination of clinical and low-density array data (*ρ* = 0.5), resulting in a correct classification of the last 4 SA samples thanks to good tie-breaking.

In view of these observations, we wondered whether addition of selected clinical symptoms could increase the accuracy of our model. The additional value of several clinical attributes was tested, using the low-density array data obtained from untreated patients with a known diagnosis: presence of psoriasis, presence of rheumatoid factors, presence of anti-CCP antibodies, elevated serum CRP values, presence of arthritis in the hands, morning stiffness above 1 hour, number of joints with arthritis, presence of rheumatoid nodules, symmetrical arthritis, X-ray erosions and synovial fluid WBC count. Out of these 11 variables, 3 were found to significantly improve the performance of the prediction algorithms: arthritis of the hands, presence of rheumatoid factors and presence of psoriasis (data not shown). In untreated patients with a known diagnosis, a 5-nearest neighbors classifier, using these 3 clinical attributes only, resulted in a diagnostic performance (3-class BCR) of 94.1%, a good performance that is not surprising in patients with clear-cut clinical presentations. Because all 3 clinical attributes are binary variables, ties subsist when determining the nearest neighbors in a clinical variables-only model. We found that addition of the transcriptomic data in the model are useful to break these ties and find the best neighbors, resulting in an improvement of the diagnostic performance up to 98.6% (Table 3 and Fig 5).

We next applied the same algorithm and resampling procedure in order to make a molecular diagnosis on the dataset available for UA data (n = 54). In this model, UA samples were probed, and all samples (UA and known diagnosis) were used as potential best neighbors. The diagnostic accuracy for UA samples was computed by comparing the predicted diagnosis to the later clinical diagnosis. Models based on 1, 3 or 5 nearest neighbors gave similar results (data not shown). Using a clinical data-only model, the 3-class BCR was 64.0% (a performance in line with the undifferentiated clinical status). Genomic data only resulted in a 3-class BCR of 47.1%, and the combined model (clinical + genomic data) resulted in a 69.9% diagnostic performance, related to the tie-breaking role played by the genomic data. Of note, when the model was set in order to discriminate future RA from the other samples (2-class BCR), the diagnostic performance of the combined model was 80.1%. By comparison, the 2-class BCR of the ACR/EULAR 2010 RA criteria was 63.8% in the same set of patients.

#### Microfluidics Qpcr

In our final experiments, we wanted to verify that similar results could be obtained using another gene expression platform. 31 UA synovial biopsy samples were tested by qPCR, in order to evaluate the expression of 95 discriminant probe sets. 30 of these samples had also been tested on the low-density microarrays. Comparison of the gene expression results showed a good correlation between the data generated by both platforms (average correlation coefficient: 0.49; range: 0.35–0.58) on the same samples.

**Fig 6:**
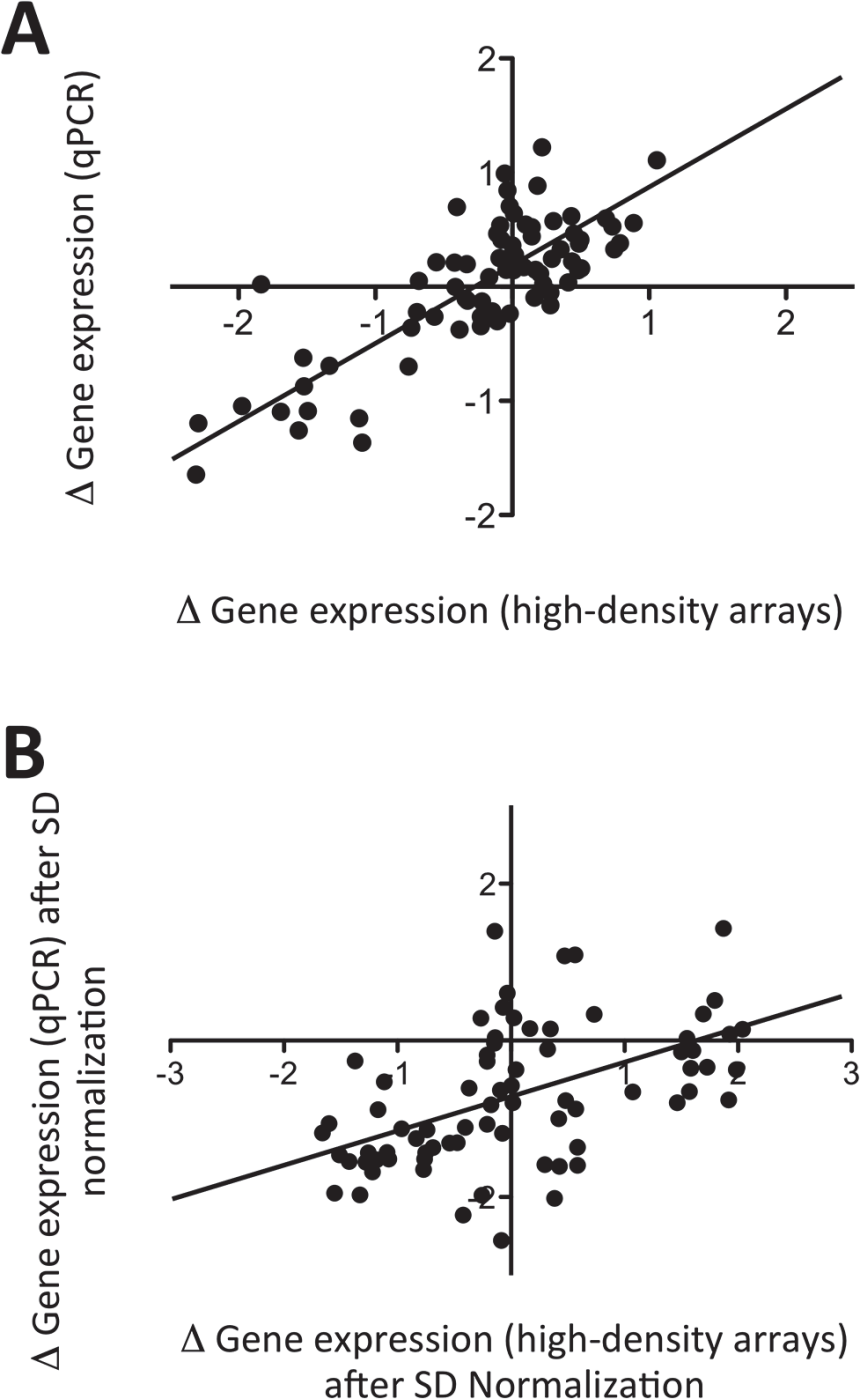
Comparison of gene expression differences between samples from patients with OA, RA and SA, using high-density arrays versus qPCR. Independent sets of samples were hybridized on high-density (HGU133 Plus 2.0 GeneChip) and analyzed by qPCR (Taqman low density array). (A) Differences in mean (log2-transformed) gene expression values between OA and (RA+SA) samples are displayed for the samples analyzed using high-density arrays (x axis) versus qPCR (y axis). (B) The same data from OA and (RA+SA) samples are displayed after normalization of each mean (log2-transformed) gene expression value by its standard deviation.

When comparing the qPCR data to the original set of data generated using samples hybridized on the high-density platforms, we also found a good correlation between the differences observed in OA versus RA + SA samples (Pearson *r* = 0.81, *p* < 0.0001). The fold-changes observed in the qPCR data were compressed compared to the high-density array data, but much less than what we had observed with the low-density arrays (Fig 6). Similarly, there was a good correlation in the differences observed in RA versus SA samples (Pearson *r* = 0.51, *p* < 0.0001) between both platforms. Again, these differences were compressed in the qPCR data, but less than what we had observed with the low-density arrays(data not shown).

**Fig 7:**
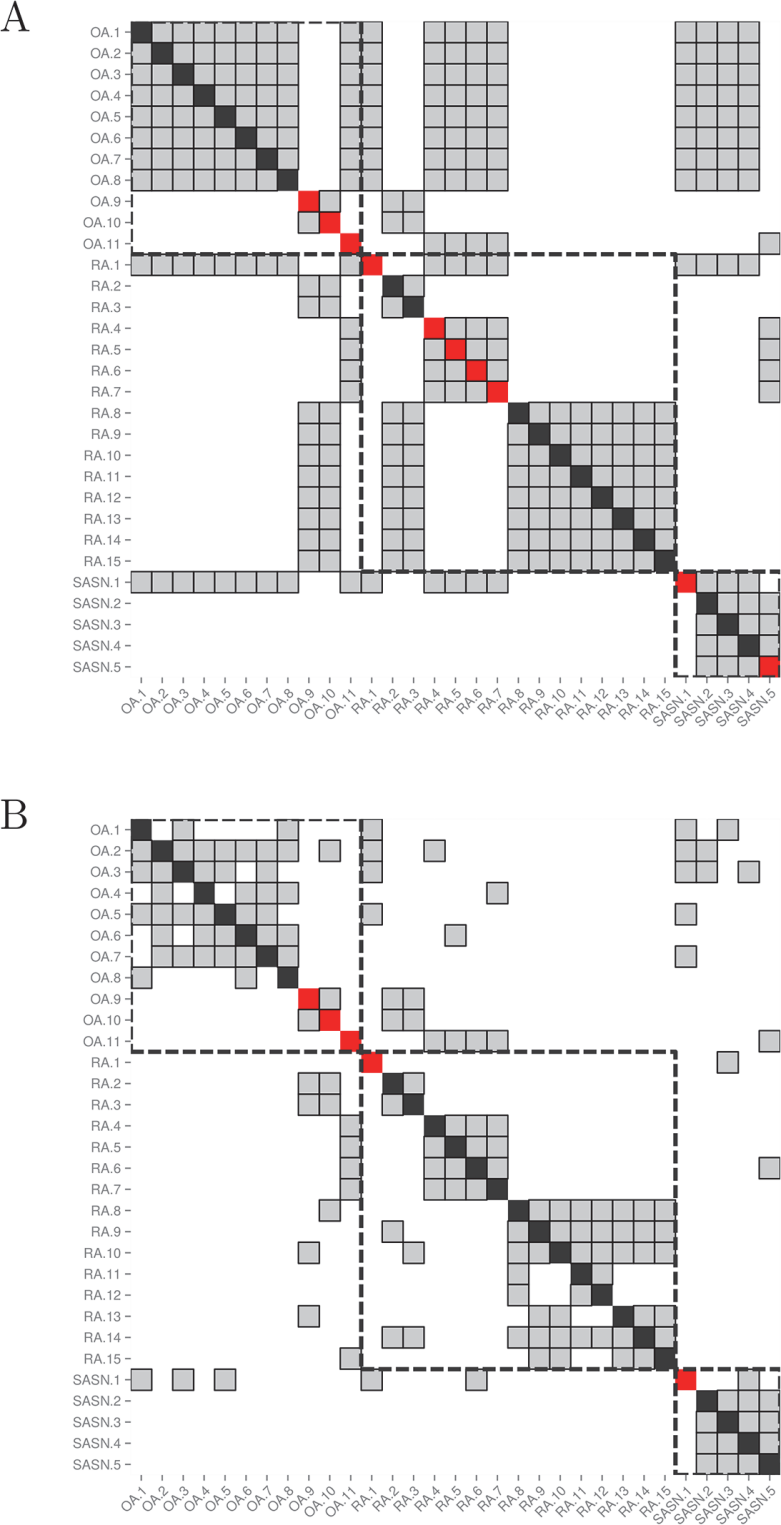
Impact of qPCR data on the determination of the nearest neighbors in UA samples. Both matrices show the nearest neighbors of each biopsy sample from the cohort of patients with UA, for whom qPCR data are available (n = 31). Each sample is represented by a column, and its nearest neighbors are greyed out in that column. The cell on the diagonal is red if the sample is misclassified and black otherwise. Samples from patients with the same diagnosis are surrounded by a dashed square. (A) Nearest neighbors are determined using only clinical data (*ρ* = 1). More than 5 nearest neighbors are displayed for each sample due to the presence of ties. (B) Nearest neighbors are determined using a combination of clinical and qPCR data (*ρ* = 0.2), demonstrating the tie-breaking effect of the qPCR data.

Accordingly, running the computation algorithm combining qPCR and clinical data on these samples resulted in a 82.3% 3-class BCR and a 2-class BCR of 90.1% for the early diagnosis of RA (Fig 7). Using the same set of samples, the ACR-EULAR 2010 criteria reached a 2-class BCR of 69.9% for the diagnosis of RA.

## Discussion

Based on high-density transcriptomic data, obtained in synovial biopsies from patients with arthritis, we designed low-density gene expression platforms in order to make an early diagnosis of RA in patients with UA. We developed an algorithm that predicts diagnosis in tested samples, by comparison to samples in a training set, using a nearest neighbors classifier. We found that the performances of that algorithm were affected by the fold-change compression of the gene expression patterns in UA samples, and overlapping gene expression patterns found in samples from patients with different clinical diagnoses. Combination of transcriptomic and clinical data in the diagnostic algorithm resulted in a high diagnostic accuracy that exceeded the performance of the ACR/EULAR 2010 RA classification criteria.

To make an early diagnosis of RA in UA patients is a « medical need » in rheumatology, in order to initiate early therapy, and thereby prevent structural damage and functional disability. In this context, the use of the ACR 1987 RA criteria may result in diagnostic delays in many patients, since several of these criteria are associated with longstanding rather than early disease. Therefore, alternative approaches were developed, or are still being currently tested, using clinical, laboratory, imaging and histological variables or combinations of them in order to improve diagnostic sensitivity in early RA. In particular, the new ACR/EULAR RA criteria are an important step forward in enabling formal identification of RA patients with early disease. However, these criteria were designed for categorization of patients to be included in clinical studies, and their use in clinical practice is still limited by sensitivity and specificity issues, as our own data also illustrate.

Recently, the development of needle-arthroscopic procedures enabled rheumatologists to collect valuable data from the synovium of large numbers of patients with arthritis. In particular, the identification of disease-specific molecular signatures in the synovium of individuals with arthritis is a step forward in the diagnostic and therapeutic approach of these patients. We therefore decided to extend these observations, using low-density arrays spotted with 100 probes, found to be the most discriminant between the tested conditions. According to our simulations using high-density array data, the adequate number of probes in order to perform the diagnostic procedure lies between 20 and 100. It should be stressed at this point that the diagnosis is carried out using a « nearest neighbors » algorithm (each sample being tested multiple times versus randomly aggregated learning sets of samples containing 90% of all other data sets). It means that the attribution of a specific diagnosis to a sample is not performed in comparison to reference values for a subset of probes; instead, a diagnosis is delivered when the gene expression pattern of the 100 probes of the tested sample neighbors the gene expression pattern of samples with this diagnosis in the learning sets.

Using low-density arrays, we found that non-inflammatory (OA) are well discriminated from inflammatory (RA and SA) samples. However, there is some level of overlapping in the gene expression patterns throughout the different conditions. It is possible that these heterogeneous gene expression patterns are related to technical issues. For instance, it appears that overall differences in gene expression between the samples are strongly blunted in the low-density as compared to the high-density array data. Nevertheless, distinct patterns of gene expression can still be recognized among the samples. Therefore, the most straightforward conclusion to be drawn from these results is that distinct synovial conditions share common molecular signatures; conversely, distinct molecular signatures can be identified in patients suffering from the same condition. In this perspective, the diagnostic performance of algorithms using these low-density array data, is not higher than 56,8%.

UA samples are also characterized by heterogeneous gene expression patterns inside the same diagnostic group. Again, compression of the gene expression differences due to technical issues plays a role, a problem that is partially resolved by the use of qPCR instead of a low-density array platform. However, our data also indicate that lower disease activity in these patients might be another source of confusion by compressing the differences in gene expression between the different conditions, since we found a significant correlation between the expression of RA-associated genes and disease activity (and disease activity is overall lower in patients with UA). Taken together, intrinsic molecular heterogeneity and compression of the fold-change magnitude in gene expression resulted in low diagnostic performances of a computation algorithm using transcriptomic data only.

The identification of relevant clinical variables, and their integration into the prediction algorithm was an elegant solution to this issue. Out of the 11 variables tested, 3 (arthritis of the hands, presence of rheumatoid factors, presence of psoriasis) proved to significantly impact the diagnostic performance of the algorithm. Our data are indicative of a true synergy between molecular and clinical variables in delivering a right diagnosis in patients with established arthritis, and the diagnostic contribution of clinical data is further illustrated in patients with UA. One should, however, be careful that the successful addition of these clinical variables in the algorithm might introduce some circularity in the diagnostic work-up. Arthritis of the hands and presence of rheumatoid factors are indeed part of the ACR 1987 criteria that we used to “certify” the diagnosis of RA in the present study. Hence, their important contribution to the diagnosis of RA might be explained by the fact that they are part of its actual definition, by opposition to the low-density array or qPCR data.

In view of these observations, the question arises whether clinical diagnosis of RA is the most adequate outcome to predict in patients with UA. Our gene expression data are in line with the observations described in the previous paragraphs of this discussion, which demonstrate that RA, as a clinical diagnosis, is an aggregate of synovial disorders with distinct histological, histochemical and molecular characteristics. Although these synovial changes lead to shared clinical and biological manifestations, hence the common diagnostic denomination, it is possible that the molecular profiles identified in patients with UA translate into distinct patterns of disease evolution and response to therapy, and, therefore, into different needs of medical intervention. Accordingly, we recently identified synovial markers of response to methotrexate, TNF blockade, tocilizumab or rituximab therapy in patients with established RA, a demonstration that a “molecular” diagnosis and characterization of arthritis can lead to specific and clinically relevant decisions [14, 15, 19, 20]. Undoubtedly, such predictive information about disease progression and response to therapy matters for the clinician as much as, if not more than a diagnostic label.

Taken together, our work demonstrates that synovial biopsies of patients with arthritis display molecular signatures that reflect the activation of specific pathways known to be involved in the pathogenesis of rheumatic conditions. The expression of these molecular signatures is heterogeneous. Clinical labels explain parts of the heterogeneity, and it is therefore possible to retrieve clinical diagnosis in patients with established disease or in patients with UA, based on the knowledge of synovial gene expression data and selected clinical symptoms. Synovial gene expression profiles are also influenced by disease activity, which is an important source of variations in molecular patterns between UA and established disease, at least in RA synovial samples. Finally, the observation that molecular patterns overlap from one condition to the other opens perspectives for a new molecular taxonomy of arthritis, if further evidence demonstrates that these gene expression signatures as such are of clinical significance in terms of disease severity or response to therapy.

## Supporting Information

S1 Fig Low-density gene expression data obtained in synovial biopsy samples from patients with a known diagnosis. Synovial biopsies were harvested in 39 untreated patients with a definite diagnosis of RA, SA or OA. The samples were hybridized on low-density gene expression (DualChip) arrays, and the gene expression values are displayed for the same targets as in Fig 2. Hierarchical clustering (Pearson’s distance) of the transcripts based on their gene expression values among the samples identifies a cluster enriched in T cell activation-related transcripts (A) in RA and SA samples, and a cluster enriched in RAS-GTPase activation-related transcripts (B) in OA samples.
(TIFF)

S1 Table Low-density microarray data. Normalized median gene expression data from 54 UA samples are displayed for all the probes present on the microarray slide.
(PDF)

S2 Table qPCR data. Raw Ct values from 31 UA samples are displayed for all the probes present in the Taqman low-density array.
(PDF)

S3 Table Probe sets and gene symbols displayed in Fig 2. (PDF)
