# Supplementary figures and images for: Heterogeneity of Synovial Molecular Patterns in Patients with Arthritis
Source: PLoS One. 2015 Apr 30;10(4):e0122104. doi: 10.1371/journal.pone.0122104 (PMC4415786; doi:10.1371/journal.pone.0122104)

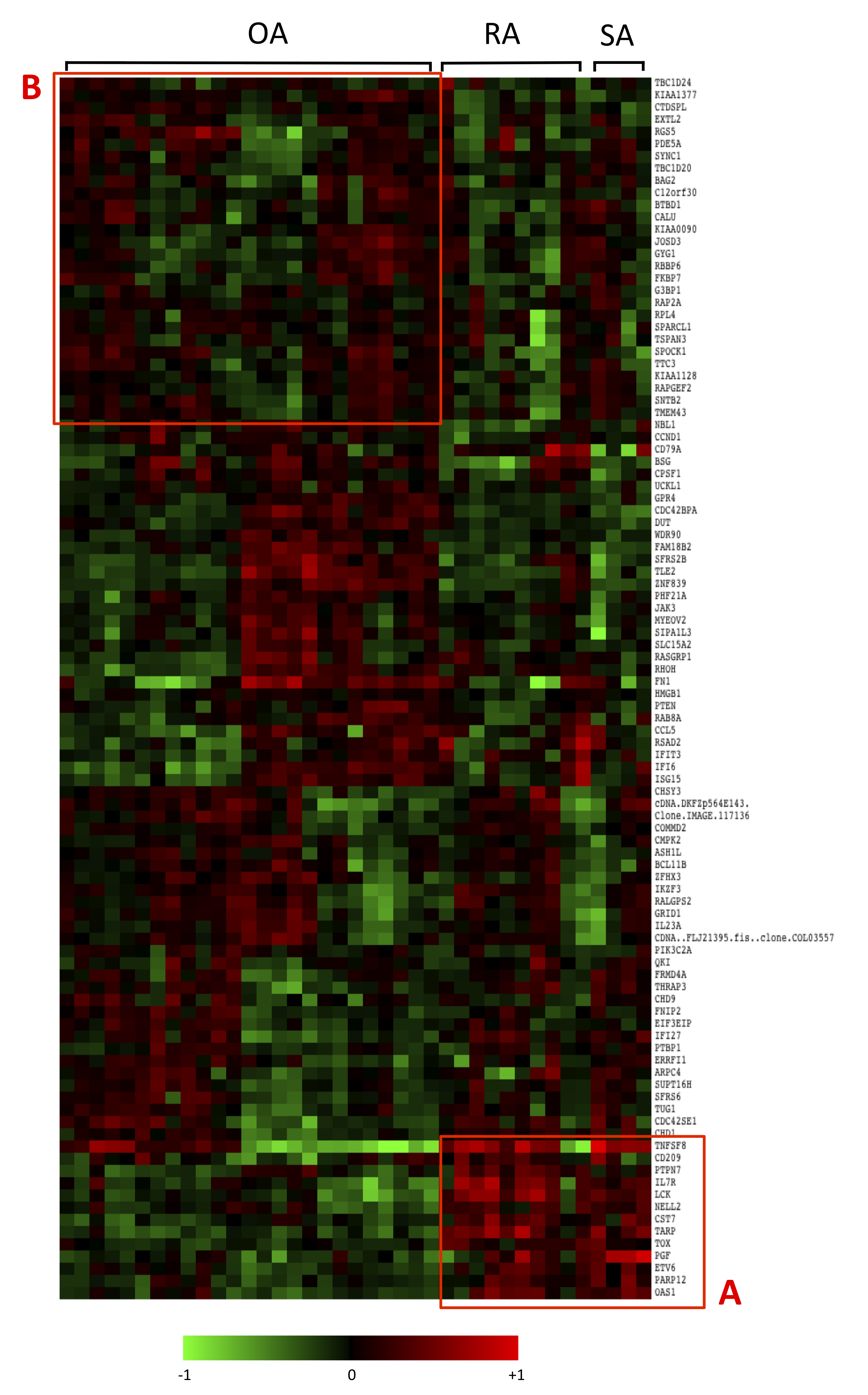

Supplement: S1 Fig — Synovial biopsies were harvested in 39 untreated patients with a definite diagnosis of RA, SA or OA. The samples were hybridized on low-density gene expression (DualChip) arrays, and the gene expression values are displayed for the same targets as in Fig 2. Hierarchical clustering (Pearson’s distance) of the transcripts based on their gene expression values among the samples identifies a cluster enriched in T cell activation-related transcripts (A) in RA and SA samples, and a cluster enriched in RAS-GTPase activation-related transcripts (B) in OA samples. (TIFF) [file pone.0122104.s001.tiff]
