# Supplementary material for: Heterogeneity of Synovial Molecular Patterns in Patients with Arthritis
Source: PLoS One. 2015 Apr 30;10(4):e0122104. doi: 10.1371/journal.pone.0122104 (PMC4415786; doi:10.1371/journal.pone.0122104)
Supplement: S1 Table — Normalized median gene expression data from 54 UA samples are displayed for all the probes present on the microarray slide. (PDF) [file pone.0122104.s002.pdf]

Low-density array, raw data

ID	FinalDx	ARPC4	ASH1L	BAG2	BCL11B	BSG	BTBD1	C12orf30	CALU	CCL5	CCND1	CD209	CD79A	CDC42BPA	CDC42SE1	cDNA.DKFZp	CDNA..FLJ21	CHD1	CHD9	CHSY3	Clone.IMAGE
DIA008	SASN	10.8907017	9.32897887	10.1145267	9.42044948	10.3417684	10.6825549	9.62274622	10.6284359	10.5875376	10.6270096	10.1917804	9.62788144	9.60203791	10.0381181	8.68182476	8.93088627	10.3933555	9.40188946	9.08774797	8.92643336
DIA060	RA	10.6337272	9.41901923	9.70814972	9.37734632	9.94724613	10.3506492	9.50517952	9.89845108	10.4314966	10.721027	9.5957407	9.8128538	9.7653295	9.7082612	9.38896614	9.47167908	9.88784849	9.50739784	9.6511771	9.58088278
DIA063	RA	11.0202789	9.90102193	10.1176035	9.23529478	10.2284403	10.567398	9.46256198	10.4468226	9.78752603	11.2305073	9.95208693	9.99270545	9.38577103	10.084326	8.79143808	9.27504364	10.7847287	9.57396762	9.13141206	9.06799352
DIA076	OA	11.5422221	9.68942533	9.99441352	9.58632207	11.0416202	10.6030092	9.47789942	10.6525106	10.3466047	11.2203164	10.2045406	9.80159019	9.41413302	10.1858869	8.94522951	9.33519359	10.95305	9.53360507	9.2194364	9.2496338
DIA083	SASN	11.2425519	9.23228078	9.63471595	9.44987549	9.31016389	10.2812092	9.01146791	10.5461066	10.4740638	9.54793148	10.1440894	9.61131228	9.11978801	9.4198966	9.28270845	9.09399234	10.5565439	9.1943042	9.32592615	8.9374396
DIA091	OA	11.1989346	9.40882908	9.47832948	9.33753281	9.90014536	10.2491037	9.14329117	10.3013613	10.2243538	10.8807098	9.61112398	9.60543518	9.23517562	10.1303109	8.95411888	9.10862277	10.5065862	9.43386668	9.08008781	8.96532077
DIA093	OA	10.6598356	9.43768262	9.86493976	9.46058087	9.95906329	10.2166783	9.31391302	10.3426465	9.94558977	10.517717	9.91693467	9.88152447	9.23498734	9.63738452	9.00939426	9.18082962	10.3345364	9.48248884	9.14598875	9.0898807
DIA096	RA	10.9600315	9.7964154	10.1681921	9.53047657	10.2157971	10.5162927	9.44998209	10.7852916	10.0996434	11.1524635	10.5620734	9.89441103	9.3913536	10.3350581	9.32374654	9.28852926	10.8742737	9.65737532	9.37626017	9.11174834
DIA103	OA	11.1054779	9.47853303	10.0455416	9.35846027	10.2986532	10.466883	9.40587431	10.6274389	10.0798892	11.0349823	10.0576658	9.42465898	9.44316592	10.1585068	9.04354131	9.03857852	10.747221	9.67256402	9.08435979	9.09717856
DIA106	RA	10.7885056	9.66568707	9.90651501	9.76882391	9.9209711	10.395328	9.66914362	10.394088	10.4668567	10.6153452	9.81936277	9.97192034	9.78753917	9.88316327	9.43117951	9.63099505	10.2665712	9.82822807	9.65952325	9.60328602
DIA126	OA	11.1650532	9.33497437	9.47854985	9.27582894	10.7889109	10.5027314	9.31737061	10.5959672	10.3321779	11.0530048	10.5056393	10.782332	9.39211183	10.0076131	8.97440345	9.22442971	10.5179855	8.9756346	9.14234961	8.72641507
DIA133	RA	11.0843273	9.66776083	9.64713223	9.43751655	10.8543537	10.3442804	9.34931048	10.3427063	10.2012019	11.271024	10.2516697	9.82228679	9.37774632	9.87404039	8.82925247	9.22331447	10.5678322	9.27232139	9.04802873	9.26994288
DIA134	RA	11.1221427	10.0157726	10.3879149	9.75165	10.8929867	10.7040108	9.78248661	10.7889474	10.0959403	11.2042832	10.4010744	9.91013077	9.77531779	10.3014413	8.98809907	9.33754116	10.9179098	9.56698869	9.22389277	9.46760984
DIA136	RA	10.5693004	9.54925221	9.8106731	9.58182308	10.2099474	10.7630885	9.70087579	10.543774	10.5397989	10.8600726	10.0375466	9.86694655	9.77664287	9.7417527	9.436269	9.54513339	10.4504521	9.63417835	9.51873059	9.46755827
DIA139	RA	10.826052	9.84923229	10.3560819	10.0093001	10.251817	10.7541562	9.59872319	11.0275737	10.5208177	10.9591326	10.3760507	10.2573421	9.58747622	10.5411516	9.2013599	9.63814577	10.8993966	9.6354737	9.35964583	9.29563011
DIA141	RA	10.4721063	9.72972543	10.0267988	9.72483728	9.94703617	10.3739335	9.53083018	10.586396	10.1870014	10.773094	10.2388359	9.99793537	9.54224312	10.1104707	9.07727638	9.44086999	10.5607651	9.49970467	9.2086312	9.37577689
DIA156	RA	11.2956049	9.83374046	9.83639107	9.72640477	10.9959265	10.6881112	9.45476243	10.592493	10.3973343	10.8241554	10.1622883	9.98832365	9.74894487	10.2420809	9.28389858	9.56787075	10.85264	9.41825567	9.40411941	9.32479795
DIA179	RA	10.9773682	9.5021506	10.2071823	9.75621942	10.4458898	10.7923687	9.68220515	10.7191663	10.7830681	10.9553993	9.81775869	10.1133241	10.0527779	9.89434532	8.85312014	9.66716214	10.3123248	9.6759311	9.20666312	9.15485542
DIA180	RA	11.0393119	9.4522594	10.1203798	9.55214606	10.5812245	10.958981	9.56572625	10.814786	10.797289	11.041615	10.0277739	10.0517135	9.92541116	9.87310588	8.72993823	9.38056117	10.4311858	9.36007756	9.05689361	9.00573336
DIA182	RA	11.0076522	9.65724457	10.2461281	9.97012961	10.5686091	10.8581671	9.80021079	10.6230836	10.7563558	11.0400538	10.1167171	10.3340353	10.2817984	10.1432001	8.72469227	9.76324182	10.3795226	9.46006107	9.15179833	9.17110201
DIA184	RA	10.8563321	9.42181525	9.96038662	9.53558291	10.1529704	10.7528551	9.70994227	10.5375413	10.5998835	10.9622555	9.78733317	9.97097393	9.88829373	9.48916941	8.89663355	9.29184432	9.8485036	9.13195971	9.07431292	9.03147492
DIA185	RA	11.1447055	9.54659912	10.4230768	9.64891701	10.6056882	10.9249297	9.6282485	10.7525432	10.9257282	11.1869226	9.83121465	10.3025297	10.1210138	9.9725569	8.69480643	9.57006712	10.3478048	9.53324113	9.07886927	9.211414
DIA186	RA	10.6017896	9.07391179	9.89242307	9.35446891	10.3572887	10.64758	9.63198582	10.3705166	10.6045977	10.7073821	9.7324765	10.0597951	9.98291028	9.62897649	8.98166364	9.47330176	10.2563823	9.36735731	9.12412857	9.13393162
DIA187	RA	10.8726922	9.28331574	10.0135748	9.34475661	10.4079122	10.6567836	9.35907819	10.4963906	10.6678013	10.9942931	9.61632053	9.93560667	9.7712134	9.73076253	8.67650169	9.25803868	10.0582061	9.01579222	8.86887999	8.87941614
DIA189	RA	10.9526646	9.48995278	10.0484586	9.56836927	10.5139102	10.9215182	9.62973864	10.4474673	10.7007065	10.9622272	9.90564189	10.0509804	9.9416636	9.64878074	8.91793591	9.4091015	10.335653	9.87745987	9.45129615	9.47644869
DIA190	RA	10.9575464	9.42418206	10.2311602	9.55310499	10.4524868	10.8371874	9.64496532	10.8111228	10.7407834	10.8081829	9.91816456	10.3342169	9.99706448	9.9103389	8.853768	9.50950602	10.2880536	9.68835894	9.34061656	9.2132748
DIA192	OA	10.5396513	9.60672899	9.92375101	9.749873	10.1165306	10.3559883	9.70076106	10.3635085	10.32917	10.7161539	9.7533792	9.92205201	9.80136443	9.56663051	9.3891102	9.68164362	9.93926298	9.84128485	9.70525818	9.60542295
DIA195	OA	10.0932384	9.55891258	9.99431658	9.66279425	10.0157198	10.4493911	9.56810894	10.3727859	10.4848485	10.711289	9.62726249	10.0004011	9.82178362	9.64281568	9.35141781	9.56273215	9.97258556	9.85799971	9.60010904	9.57863006
DIA196	RA	10.2989959	9.64658432	9.73089882	9.84911084	9.91517058	10.0007873	9.53602374	10.006134	10.6074953	10.4838145	9.77658382	9.94539822	9.83446433	9.73285983	9.50893792	9.72271172	9.7994374	9.82956666	9.76971424	9.68204571
DIA202	RA	10.8803726	9.4475203	10.2678419	9.69910693	10.5492932	10.9092085	9.52788132	10.7901709	10.9256377	10.8666043	9.68218915	10.0517208	10.0126415	9.79784864	8.9022307	9.46515592	10.294062	9.55512969	9.13612896	9.08190875
DIA208	RA	10.8588228	9.45946998	9.91771291	9.5329408	10.1473277	10.6632127	9.44738272	10.6068134	10.4712921	10.9476683	9.75320852	9.91231083	9.58007489	9.56629658	9.17230865	9.44893785	10.166366	10.0301591	9.62935333	9.60204503
DIA210	OA	10.9738223	9.40100933	10.0021349	9.34894976	10.6380673	10.8446303	9.36402248	11.1726078	10.6970324	11.3785813	9.61256541	9.78249635	9.77406419	9.48338151	9.22965618	9.38026329	10.0388206	9.46914872	9.30756246	9.24840643
DIA215	SASN	10.9581566	9.48947689	10.0224712	9.72159231	10.6253921	10.8694777	9.56186587	10.8177456	10.7692431	11.1397572	10.3847253	10.0340966	9.94167263	9.54429622	9.19864168	9.55456273	10.1886198	9.98890883	9.6848821	9.67543739
DIA231	SASN	10.6961925	9.64864309	10.2005098	9.7635187	10.4295824	10.7423002	9.66923192	10.5085741	10.6459204	10.7167149	9.57975861	10.1697096	10.1297063	9.6350154	8.6868862	9.59866753	10.4212186	9.72538197	9.06893038	9.28258493
DIA235	RA	10.8867434	9.5833294	10.2549856	9.5557512	10.3359526	10.9220057	9.74722332	10.7296633	10.7020657	10.9482561	9.89182318	9.98535386	10.0359383	9.47118536	8.66938713	9.51736434	10.4140375	9.40104684	9.03830337	9.0555894
DIA236	SASN	10.8314002	9.56282626	10.1596754	9.62059053	10.5071019	10.7497619	9.44182947	10.7916447	10.7367501	10.8339181	10.3290835	10.1449718	9.95394853	9.71156446	8.94186737	9.52097535	10.2392082	9.33929265	9.19720045	9.04561375
DIA285	SASN	10.9585097	9.65710893	10.3044607	9.75102729	10.4878191	11.0413436	9.69006527	10.9155312	10.9220419	11.0488107	10.3172779	10.2219915	10.0815448	9.73786083	8.79125213	9.46570386	10.3308045	9.36214151	9.00419799	8.93012126
DIA286	OA	10.7673173	9.26791012	9.826827	9.48447642	9.72636143	10.3937558	9.33158221	10.2860786	10.153152	10.2353688	9.61700121	9.74416413	9.68058345	9.38528422	9.00030354	9.37843523	9.88928473	9.52875754	9.35975458	9.38416119
DIA289	OA	10.7335258	9.23626597	9.61467366	9.3921078	9.82468756	10.1165473	9.27793857	10.2386015	10.2474612	10.6491863	9.21042678	9.55798969	9.45584927	9.31657005	8.98800757	9.24198469	9.73935312	9.70542697	9.71381023	9.77334734
DIA290	RA	10.9326355	9.2465383	9.50909901	9.42964093	9.69772646	9.99396256	9.35698829	10.0498649	10.0323149	10.1096257	9.29249692	9.43579199	9.42046546	9.44406131	9.2545817	9.21984838	9.49965839	9.68263518	9.40604317	9.49408519
DIA292	RA	11.0408953	9.39982098	9.41976505	9.6070485	10.5525333	10.5006515	9.2199349	10.5296443	10.9488338	10.9358728	9.87743726	9.87252708	9.59266386	9.4456826	9.14580848	9.3409651	10.0736242	9.20254526	9.15267017	9.14609476
DIA297	OA	10.638125	9.66421007	10.0463232	9.64411442	10.9470977	10.9081155	9.59889919	10.8198912	10.8479177	11.1958005	10.007037	10.1538931	10.0986963	10.12031	9.13341162	9.60540441	10.462756	9.45578864	9.43174306	9.37537475
DIA299	OA	10.9328445	9.0509753	9.22506402	9.2694893	10.751283	10.7841029	9.06051585	10.5205997	10.7503967	11.1419017	9.15710896	10.0298699	9.95659673	9.66442518	8.94590042	9.29431795	10.2944912	9.20290062	8.97953649	8.94861125
DIA305	SASN	11.0411082	9.50497195	9.71845375	9.76700094	10.7335949	10.6485742	9.44401068	10.7940275	10.7585781	10.9533228	10.3338443	10.1213592	10.0441476	9.73439847	8.79414074	9.44142286	10.4369798	9.09267718	9.0265029	8.87128266
DIA306	OA	10.9275248	9.529199	9.9525934	9.61576576	10.7661066	10.7192604	9.4644578	10.6384275	10.6704436	11.0499877	9.76287354	9.91631366	9.96983524	9.75569245	9.07786461	9.38995191	10.2336833	9.38922906	9.34388584	9.1418611
DIA308	OA	10.4738419	9.31534555	10.0429525	9.35473303	10.2480862	10.9227584	9.57792088	10.7076414	10.7312426	10.7541455	9.75593168	9.85493802	9.73733356	9.31250541	8.90418692	9.27960015	10.3883306	9.13813933	9.11350664	8.97802886
DIA309	SASN	10.5029117	9.35035842	10.1584815	9.39662442	10.1269671	10.9123457	9.76860775	10.7478256	10.6926364	10.5059383	10.4275265	9.74802505	9.74102128	9.36921267	9.02915336	9.35152677	10.3077312	9.26373778	9.1326828	9.05837836
DIA321	RA	10.9336197	9.70378146	10.1924195	9.45447122	10.8510351	10.8804172	9.87073661	10.7138265	10.6452537	10.9824042	10.2873884	9.95080468	9.98341773	10.054455	8.50699964	9.05648421	10.6221329	9.58976391	8.91221979	9.18097487
DIA325	OA	11.0063614	9.03962425	9.63873531	8.6829542	10.8806872	10.8645276	9.36285475	10.6780423	10.6717863	10.9353823	9.86668031	9.67126849	9.70209381	9.77330627	8.34254443	8.79417426	10.417084	9.22781006	8.87527646	8.77750586
DIA348	OA	10.9089327	9.28740071	10.071644	9.35866516	10.5638145	10.7332263	9.39647491	10.7643665	10.6659679	10.973807	10.1558815	9.65964517	9.87069707	9.93019598	8.2504381	9.01521311	10.1245922	9.31125994	8.75721709	8.55393327
DIA349	OA	10.895837	9.41371115	9.93188515	9.37234285	10.608373	10.7050882	9.42608693	10.7452031	10.6302942	10.8825958	9.74407156	9.71719787	9.8046107	9.89069587	8.37438781	8.96831263	10.1572714	9.28373474	8.82870333	8.81677796
DIA354	SASN	10.8371408	9.50462197	10.1783134	9.53495021	10.5869768	10.7289933	9.52439884	10.7874442	10.7188898	10.8394266	10.1138417	10.0550515	9.98165035	9.97338894	8.46826942	9.01218096	10.4050603	9.477145	8.94222988	8.75280932
DIA356	OA	11.0437219	9.50290214	9.99234121	9.45408195	10.8039687	10.7409662	9.41017976	10.9969609	10.5770496	11.0562771	10.4259281	9.86487913	10.125197	10.1113783	8.36848502	8.95198493	10.2743741	8.98619493	8.71232615	8.63092853
DIA358	OA	10.9050746	9.4772541	10.2576614	8.80946523	10.6205503	10.9574758	9.48955153	11.0504644	10.7985905	11.1397907	10.05101	9.80795228	9.9483633	9.70147693	8.39598491	8.84533788	10.2646169	9.17499847	8.86014571	8.70308112

CMPK2	COMMD2	CPSF1	CST7	CTDSPL	DUT	EIF3EIP	ERRFI1	ETV6	EXTL2	FAM18B2	FKBP7	FN1	FNIP2	FRMD4A	G3BP1	GPR4	GRID1	GYG1	HMGB1	IFI27	IFI6
9.17328603	9.39514405	9.67997993	9.98600059	9.65818151	9.50891625	10.8844661	10.99928	10.0461025	9.76872333	9.38397881	10.0063327	10.9970597	10.4743969	9.89397731	10.8744764	9.69858544	8.85310443	10.5276015	10.7930322	10.6962052	10.3913491
9.7051717	9.49954759	9.88855728	10.0700953	10.1575775	9.61337982	11.060465	11.777012	9.79438638	9.75878038	10.0036699	9.8301597	11.8242156	10.2923456	10.0482271	11.2614253	9.76747905	9.58955492	10.0577422	11.1923231	10.3881793	10.2821904
9.73414745	9.16123523	10.1223394	9.69573119	10.3110678	9.3295111	11.5538642	11.6290698	10.0642162	9.80352823	9.70718494	9.84710887	11.0135458	11.1359853	10.5299665	10.8593725	9.72875729	9.10290311	10.5763743	10.8693968	11.6657934	10.6881837
9.38546609	9.31217516	10.2883005	9.67714265	10.0344862	9.29364092	11.2090084	11.7119338	10.0042277	9.86101474	9.59349062	9.63246022	9.9994907	10.9594671	10.3326104	11.2218695	9.6512692	9.21622907	10.3318647	11.0810936	10.8212189	9.92056624
9.23004211	9.16513992	9.9439479	10.5913788	9.67488034	9.15299333	11.2770623	10.8486609	10.0303604	9.546325	9.16834653	9.50503974	10.7543353	10.6293034	9.95132302	10.8881287	9.35210908	8.96339705	10.2014134	10.8859037	11.2727518	10.1189157
9.31642686	9.16039089	9.76992717	9.55103508	9.60749357	9.02997152	11.2668803	11.6796606	9.97582481	9.40696147	9.35391317	9.40930418	10.2370781	10.4658232	10.0142518	11.0753096	9.37670286	9.04664536	9.95674957	10.8254951	11.1969203	10.0963952
9.25577929	9.22885916	9.81652448	9.4299754	9.89638193	9.27157682	11.2155442	11.5569967	9.72490504	9.61252432	9.47596296	9.70538353	11.0325454	10.7396847	9.97405348	10.6210835	9.62409236	9.10718104	10.128522	11.0815908	10.9688236	10.009172
9.43209545	9.13540444	10.2039706	9.93113395	10.0033406	9.14072237	11.4472289	11.8084193	10.2915863	9.81622356	9.70065083	9.60227321	10.2568101	11.166336	10.2964592	11.2424091	9.84173409	9.00794033	10.2125135	11.2866233	11.1675125	10.2209236
9.33374077	9.3566179	10.1679503	9.51524071	9.96591645	9.51928315	11.2872753	11.5864179	9.80823908	9.89865117	9.5249934	9.65371901	10.4821564	10.769512	10.3988439	11.1290483	9.49932896	9.02648298	10.1126894	11.1810021	11.0106378	9.92726097
9.73136434	9.56884247	10.1124611	9.99406926	10.0048103	9.76312779	10.9542833	11.4130692	9.90901807	9.82642332	9.98493361	9.90602584	11.3988111	10.4244885	9.99550843	11.0711723	9.74862137	9.49208732	10.3041228	11.0089635	10.7673078	10.6201254
9.25714866	9.10749184	10.2501379	10.6002926	9.77301075	9.14439578	11.4742777	11.6376051	10.3661001	9.65120313	9.47933184	9.80923998	10.5982229	11.1298019	9.911262	11.2715223	9.54291934	10.1061992	10.3750443	11.1516495	11.2637456	10.3712159
9.26814542	9.27003098	10.1020093	9.61813173	9.82508477	9.10476036	11.1679326	11.0816768	9.92792649	9.52016739	9.38160761	9.3923125	10.3066891	10.8772931	10.3857493	10.992019	9.541114	8.95856746	10.1388203	11.0203243	10.9410094	9.87114458
9.51262726	9.30148428	10.5861095	9.6514342	10.40327	9.47584854	11.2143263	11.4222786	10.2889648	10.1419055	9.72596935	10.0116616	9.9905697	11.0940811	10.6532069	11.1259324	9.78435605	9.28798033	10.3552013	11.119431	10.9772113	9.99891634
9.64092193	9.48047395	10.073625	9.7468336	9.80719156	9.673607	11.1286401	11.7036801	9.83851875	9.69001509	9.85857642	9.88991261	11.7435316	10.6847476	10.2575361	11.4896636	9.83107035	9.54667431	10.5618567	11.1886458	10.769398	10.2405546
9.74307988	9.61617566	10.2138228	10.4908858	10.1605021	9.47021934	11.4618569	11.6709842	10.4128458	9.92662997	9.83467928	9.83445393	10.6253348	11.0030516	10.2029456	10.9194987	9.92035491	9.56462707	10.640997	11.2375922	11.3981612	10.5100892
9.57753315	9.37165933	9.83004851	10.0391867	9.93375289	9.43197708	11.3829152	11.5540013	9.90317782	9.92890669	9.6026237	9.76955061	10.8460573	11.0442328	10.0624967	10.7679777	9.66743127	9.21452282	10.2208613	10.9332824	11.2592769	10.0110173
9.80058245	9.50498143	10.3056755	9.92329202	9.93101532	9.33041821	11.1994665	11.7979689	9.97655117	9.73876917	9.66902713	9.74270675	10.3068079	10.5879341	10.0049372	10.7168885	9.57330689	9.45133779	10.0019772	10.9683225	11.0806637	10.6562396
9.63522469	9.15057734	10.3781932	9.97343094	10.0323466	9.80834501	11.2263864	11.4333612	9.81100362	9.86603843	10.1373724	10.0337792	11.5406374	10.8293254	10.0926152	11.1063899	9.79800535	9.44324591	10.5377751	11.2332361	10.8444723	10.4703349
9.47018368	9.07145703	10.2611433	9.98035819	9.75570151	9.59764525	11.2555688	11.599678	9.83584982	9.64485541	10.0022837	9.75870785	11.7580386	10.7639217	10.006983	11.383709	9.64929638	9.29704167	10.6384908	11.2890229	11.2211222	10.7943117
9.84235216	9.15714392	10.5439631	10.1855372	10.0953592	9.83782011	11.154339	11.2912327	9.86536914	9.85566496	10.3324744	9.88522405	11.3513801	10.8223611	10.1843969	11.0836552	10.0377142	9.54268294	10.6122925	11.1378242	10.6909997	10.4342681
9.36447763	9.02838736	10.0247668	9.87189216	9.63680283	9.34452052	11.2570956	11.7210578	9.51310997	9.28333235	9.87356453	9.51419039	11.7822101	10.370184	9.64960589	11.4123581	9.34500291	9.09676444	10.2200014	11.3807855	11.2003352	10.7066324
9.76654992	9.03814067	10.5257557	10.1852863	10.214456	9.91513561	11.4120877	11.6193556	9.82898608	9.91284244	10.2727211	9.97130859	11.738614	10.8881436	10.054835	11.3834038	9.63180932	9.28424757	10.6690981	11.3682255	10.9488208	10.5955976
9.61090202	9.09187566	10.2354266	9.85960669	9.79540569	9.58110467	11.128859	11.3116208	9.52102503	9.53864747	10.0783418	9.64483464	11.435404	10.5965346	9.99893536	11.0533629	9.7180891	9.2924961	10.3101259	11.0646553	10.9698885	10.7844661
9.2991196	8.85457838	10.083188	9.93311882	9.68388761	9.48627197	11.3108075	11.5291621	9.66388269	9.41893043	10.0281849	9.65248735	11.5882714	10.5539487	10.0945794	11.1658537	9.62436823	9.29644807	10.5413551	11.143258	10.9794258	10.6722438
9.50320064	9.15840181	10.2530201	9.96623864	10.1904782	9.66699713	11.2489321	11.0844101	9.66235239	9.79229554	10.0419626	9.66859365	11.4964651	10.6458361	10.198837	11.1946886	9.64422382	9.20854083	10.5575456	11.2019405	10.5102017	10.3202439
9.57966461	9.2688298	10.38368	10.2040699	10.0477509	9.68908682	11.1338834	11.1866104	9.92824354	9.94635128	10.0973385	10.0828347	11.2921945	10.6177406	9.97009383	11.1716987	9.62098517	9.21303129	10.5936088	10.985085	10.813676	10.7601386
9.73566694	9.57846494	10.2016807	9.97304199	10.0066749	9.67360125	10.8019458	11.05249	9.73148788	9.71066639	9.92791227	9.76695761	11.3545827	10.2290687	9.84042661	10.942607	9.84044278	9.68757578	10.0919334	10.858261	10.3659516	10.0495183
9.6462345	9.50326119	10.0014765	9.87991227	10.084303	9.63457157	11.0344111	11.4195579	9.75245907	9.78594111	9.96659832	9.82372029	11.6136812	10.4373162	9.92894002	11.1243331	9.74267625	9.5780248	10.1675704	11.1564295	10.6119308	10.1645724
9.80223413	9.60255211	10.1598756	10.0666596	9.99593913	9.65494972	10.7662662	10.3445539	9.77190748	9.71174414	9.98669683	9.7686923	11.4392279	9.95252411	9.78988409	10.727535	9.76009305	9.67469059	9.87661671	10.6760623	10.4441319	10.0266345
9.59385303	9.13134257	10.3536873	10.0582804	10.0065023	9.70539049	11.1984806	11.3611101	9.83022607	9.7935162	9.994958	9.86620759	11.4547503	10.5840374	9.97054466	11.1664054	9.55385551	9.2700527	10.6006829	11.2273747	10.9466342	10.5620059
9.54580288	9.41966656	10.1783074	9.88430013	10.2869398	9.44480945	11.1804515	11.4641336	9.64058972	9.6541581	9.79018137	9.82099496	11.6317343	10.4802702	9.94902014	11.0018686	9.57142116	9.33956083	10.1456433	11.0833918	10.8101693	10.4053008
9.44204864	9.29750782	10.0907601	9.64829478	9.87323616	9.48660297	11.2762647	11.6064378	9.66399465	9.84281297	9.8509983	10.0381678	11.8114214	10.7744835	9.66367318	11.5843829	9.66500024	9.32310403	10.3545401	11.2354404	10.3825833	10.1708704
9.61063598	9.45161369	10.2376339	10.0803975	10.2769872	9.63038125	11.0686785	11.3793899	9.98369022	9.78468398	9.99336226	9.82308587	11.472674	10.5067328	10.0762173	11.2267404	9.82023853	9.42102476	10.4398189	11.0229714	10.6966925	10.388482
9.66089922	8.95484356	10.3570294	9.93102431	9.92650559	9.77456589	11.0960689	11.1251042	9.71628396	9.64555684	10.1291652	9.8876727	11.3026275	10.5256099	9.99983765	11.0316455	9.67126508	9.24284256	10.3164887	11.0363118	10.4967375	10.1032273
9.59277282	8.8946188	10.2400278	10.0069229	9.91638637	9.63760713	11.2014603	11.4813946	9.72075493	9.84805364	10.110616	9.90671835	11.5580005	10.7623186	10.0117808	11.2058796	9.53927519	9.15597263	10.5672114	11.1689494	10.9618553	10.5006677
9.67756794	9.04107091	10.2210205	10.0990394	9.92631133	9.69941678	11.0682224	10.9238181	9.93637786	9.81235541	10.0686516	9.86434442	11.3241043	10.5010423	9.89153348	11.1931988	9.68448433	9.28592033	10.4497316	11.0383007	10.7911964	10.4674664
9.59904378	8.94712183	10.2769831	9.98494421	9.85200076	9.51718123	11.2030036	11.390843	9.64276108	9.55622306	9.99466774	9.8095174	11.5418179	10.6367194	9.88584544	11.2793604	9.53124916	9.00277986	10.5272449	11.1882857	11.0293475	10.4812521
9.50674442	9.26869057	10.1241066	9.81143818	10.047446	9.61323591	10.9623465	10.6798382	9.62082566	9.77600239	10.1249498	9.83173498	11.1878923	10.4732963	9.95957326	11.121418	9.52856538	9.4347607	10.6911852	10.9503271	10.656567	10.4322362
9.49804332	9.05010427	9.87553174	10.4078214	10.5813474	9.37265387	10.969088	11.4346679	9.94253585	9.85160834	10.0722007	9.95747866	11.5793535	10.4939577	10.3343876	11.1316508	9.50031649	9.32926374	10.1521805	11.0551236	10.6914	10.3015961
9.4284611	9.24571341	9.67443585	9.98176562	10.3214571	9.4947729	10.6436036	10.9561386	9.53970608	9.68112108	9.78865549	9.57318506	10.8937145	10.1728558	9.99241361	10.9208071	9.46789848	9.39780271	10.1323067	10.7505334	10.3070219	10.0776423
9.21620689	9.16540923	10.1222015	9.51561148	9.54887552	9.37994367	11.2139833	9.88607657	9.71733261	9.2064115	9.99978949	9.33996716	11.8530915	10.238879	9.61298907	11.6553201	9.69260029	9.16934273	10.220289	11.397616	10.928608	10.6945181
9.66775071	9.37307161	10.4056818	9.99718375	10.0685548	9.75849618	11.2883245	11.7100341	9.83741991	9.88785386	10.1593119	9.86244631	11.8326566	10.8474935	10.0879422	11.4638154	9.84764543	9.35480433	10.4997801	11.3941272	10.5133425	10.5563002
9.50682166	9.02201325	10.2715186	9.99014629	9.85603605	9.54511885	11.3213354	11.609579	9.71085645	9.81711688	10.1263646	10.0736212	11.6068528	10.9212937	10.0281856	11.4081448	9.79469933	9.26601036	10.6143711	11.3208585	10.5956466	10.7510834
9.61282062	9.14969234	10.3629532	10.1529064	9.75088921	9.61238865	11.1931869	11.2416523	10.1348531	9.61452534	10.1135146	9.68816824	11.5299387	10.6337679	9.80466052	11.3203121	9.88196962	9.28297887	10.7295058	11.2871108	10.8173606	10.8695625
9.56553862	9.25532167	10.2175833	9.90225229	9.94394817	9.67037036	11.0989813	11.3520349	9.8015164	9.95042711	10.1092267	10.1631455	11.3634447	10.691398	9.92752143	11.1619715	9.91861375	9.39326822	10.4611283	11.1450466	10.5482628	10.6696572
9.43913957	9.17921604	9.90004219	9.87146974	9.71715565	9.59555872	11.1710158	11.4281664	9.69071894	9.92997997	9.8584155	9.86689579	11.4970963	10.8744698	9.71460525	11.250564	9.88583238	9.18000474	10.6531541	11.0656478	10.1793465	9.973892
9.22689353	9.23206279	9.78221813	9.88691402	9.52230625	9.46232068	11.0630828	11.3744755	9.63737492	9.76639682	9.67324282	9.73256831	11.382674	10.5335819	9.85873088	11.2452444	9.78372394	9.24086799	10.7142474	10.9887543	10.2869075	10.0135358
9.43428818	9.21224545	10.2270937	9.78491206	10.1273189	9.557577	11.1429277	11.2341938	9.95474261	9.9042272	9.9248042	9.91362731	11.2503168	10.6985353	10.3145214	11.0514618	9.95021474	8.94984614	10.7170388	11.1081327	10.7233075	10.5586305
9.12800739	8.7303791	9.90328998	9.67505885	10.1429265	9.59307026	11.223468	11.0808961	9.79715554	9.96399108	9.63333862	9.86623336	11.3439326	10.7933364	10.3185635	11.1269266	9.63923926	8.83142114	10.6321607	11.1191766	10.7177739	10.6546812
9.16803317	9.01601501	10.0214421	9.76846648	9.78114746	9.48030031	11.0411195	11.1143659	9.92455834	10.0052307	9.83858874	10.2568208	11.2058919	10.5787903	9.89520325	11.0356039	9.89197882	8.90127056	10.535656	10.9566453	10.6438078	10.5123894
9.27421628	8.93305455	10.0310605	9.57046998	9.93335173	9.56042168	11.0433195	11.1465069	9.49206683	9.72451449	9.80946751	9.86025868	11.2544729	10.6821966	10.2031308	11.0608813	9.64371277	8.87774981	10.5137807	11.0810834	10.8317553	10.7094007
9.4230027	8.95645555	9.98503593	10.0892358	9.80904112	9.53702634	10.9987187	11.0234181	10.0582377	9.65250262	9.8473909	9.77267292	11.1533419	10.4954834	10.0900828	11.0744693	9.78965955	8.93675659	10.6114594	10.9307079	10.7822512	10.7160909
9.30026858	8.77017512	10.1730248	9.58445228	10.048975	9.46009565	11.112785	11.1845625	10.0366618	9.94300016	9.80508568	10.0794532	11.2455134	10.5747467	9.90508842	11.0421815	9.85334273	8.71066094	10.622161	11.0113529	10.7407626	10.6218907
9.3675401	8.71238838	9.96102554	9.82998064	10.0372338	9.53222736	11.14592	11.3062989	9.76962479	10.0475574	9.91890602	10.0970779	11.2910028	10.5972844	10.1097388	11.1791932	9.55647797	8.76774506	10.5552303	11.0450761	10.885724	10.7993788

IFIT3	IKZF3	IL23A	IL7R	ISG15	JAK3	JOSD3	KIAA0090	KIAA1128	KIAA1377	LCK	MYEOV2	NBL1	NELL2	OAS1	PARP12	PDE5A	PGF	PHF21A	PIK3C2A	PTBP1	PTEN
10.5980243	8.93648617	8.86001196	9.9239619	9.81236656	9.13460389	10.0933832	9.70882445	10.4856097	9.49590864	9.91319288	9.46869956	10.4770618	9.25133532	10.4874929	10.2232127	9.7423754	9.99556668	9.57169245	9.24328995	10.5525482	9.55175955
10.1107122	9.54282567	9.60115766	9.45536082	9.97243633	9.63635432	9.83112241	9.7468279	10.5002027	9.80752185	9.9434884	9.8089033	10.7577946	9.62075702	10.0397245	9.91237399	10.0913422	9.61478534	9.97495931	9.62867809	10.6688428	9.487997
10.8671278	9.22712097	8.86117283	8.86147402	10.819546	9.13125025	9.59676823	9.66551327	10.9038309	9.88912141	9.44081962	9.43484383	11.2313334	8.8965579	11.0523944	10.960817	10.8392578	9.91289201	10.0899507	9.27056074	11.4804385	9.00286068
9.98628846	9.2442046	8.98735366	9.04637479	9.3770787	9.21451846	9.64224767	9.70340295	10.6275657	9.72028133	9.45717937	9.30939337	10.7874066	9.01684251	10.4311721	10.1748645	10.3260005	9.68770149	9.90108502	9.3555834	11.2926739	9.18052455
9.89940335	9.4178526	9.00208392	9.74724103	9.35017119	9.08165095	9.93832107	9.53936752	10.4857728	9.39662083	10.4410007	9.10487708	10.5736543	9.70473507	10.9549322	10.2112615	10.1734723	10.4964712	9.49022283	9.24683105	11.0945498	9.09222542
10.6225589	9.04556675	8.9364812	9.54832964	9.40294756	9.19438634	9.35116879	9.47823531	10.3206013	9.28772661	9.82179924	9.14181487	10.6411187	9.13297632	10.5541646	10.4696222	9.65899659	9.99431971	9.52996033	9.28702604	11.0362716	9.00711831
10.2158006	9.11687928	9.08684715	9.07840795	9.41085148	9.11722788	9.48281965	9.69279811	10.3681108	9.71228859	9.23927763	9.11654562	11.0415332	9.0567622	10.0841685	9.83336508	10.1932868	9.78535813	9.59746881	9.25778143	10.961659	9.07253686
10.3733804	9.13157148	9.12563702	9.37039637	9.09276553	9.37677464	9.72101008	9.71833769	10.7550703	9.68207405	9.58262846	9.06164253	10.9222351	9.10156757	10.5442065	10.2733315	10.4336126	9.87181276	9.86188025	9.44087309	11.0695945	9.01549978
10.5092537	8.98603226	9.00123593	9.05451401	9.40581582	9.04658333	9.61712518	9.57630419	10.6088306	9.71225687	9.45125601	9.02032024	10.7418514	9.04413472	10.6438718	10.1073259	10.0535462	9.60554868	9.58051263	9.5397913	10.953405	9.09083368
10.3269249	9.49363001	9.51355238	9.57772454	10.1254498	9.71390159	9.97593495	9.87377991	10.5581387	9.85498798	9.88794309	9.72089443	10.6924843	9.74553362	10.1923313	10.0802202	9.99728962	9.51409111	9.88970064	9.60261943	10.6578747	9.62053395
10.3785879	8.89918269	8.95116459	9.65415766	9.60150518	9.18835978	9.58445089	9.62393241	10.4218682	9.46228904	10.2785389	9.00841892	11.0030336	9.22603115	10.4123269	10.5549096	9.89699634	10.4542994	9.68422798	9.20288314	11.4002121	8.87985392
10.2482207	8.83606971	9.26215174	8.95451688	9.41308198	9.30339873	9.65293523	9.53911148	10.5563479	9.4637946	9.50400402	9.11839287	10.6645915	8.88595171	10.5674803	10.0855877	10.4736668	9.6989825	9.74851845	9.44328235	11.240929	9.20169434
10.4325973	9.05346864	9.0007685	9.00574886	9.45526494	9.17784837	10.0185682	9.79275432	10.8183961	9.88316685	9.4632164	9.42264426	10.9383119	9.0037389	10.6122649	10.2804918	10.543485	9.91995107	9.92490745	9.2544308	11.1627176	9.09083885
10.2504355	9.48906056	9.47876697	9.49726392	10.0176096	9.53891372	9.99069628	9.87637706	10.7333427	9.67749156	9.69384865	9.60688299	10.6782988	9.53569098	9.84091518	9.80220088	9.82848208	9.45601547	9.64145239	9.46729725	10.7451691	9.49390779
10.7335789	9.49572651	9.2736139	9.95952793	9.68101997	9.50426151	9.90977425	9.86137407	10.4826053	9.76959681	10.2499674	9.51336668	10.6999975	9.59478823	10.7706227	10.5810281	9.75259051	10.3642041	9.85589627	9.28734619	11.1345057	9.1733681
10.0726385	9.34870272	9.11527394	9.54441657	9.44345367	9.27458838	9.78288824	9.67760387	10.488855	9.51611599	9.81233892	9.45287023	10.6808163	9.42638838	10.5728278	10.3330584	10.1418178	9.97693439	9.8562062	9.19022759	11.1888558	9.0519098
9.96524039	9.39707116	9.29970312	9.40976192	9.78234224	9.40470661	9.72720602	9.56125082	10.3407901	9.47243826	9.43168904	9.51071709	11.3688035	9.70300373	10.4779115	10.0929514	10.3043502	9.96700479	9.89712551	9.53357751	11.0970351	9.49399869
10.3262291	9.2859167	9.38651639	9.14856337	10.1863357	9.48648776	9.91493462	9.83311362	10.8353198	9.86580809	9.40184086	9.74491997	11.0807805	9.19773104	10.2769267	10.0800377	10.3958019	9.64142161	10.0767532	9.45038148	10.8436646	9.54918843
10.6144463	9.14913181	9.20287485	9.1554068	10.0922885	9.3859302	9.88563016	9.67528845	10.8977361	9.58487838	9.4488689	9.6478803	11.1796198	9.10311792	10.5456012	10.4214744	9.94243486	9.30196514	9.9932256	9.28636006	10.9877778	9.40296824
10.6072224	8.69841446	9.48986949	9.07752031	10.1844429	9.68384581	10.032355	9.85748871	10.8166368	9.98173908	9.53977401	10.0074732	10.9408235	8.88718667	10.1351432	10.1085316	10.271924	9.63705839	10.3655054	9.25260359	10.8899876	9.65845113
10.7311548	9.08706688	9.08836407	8.95980983	9.9299115	9.18549653	9.66943945	9.43772817	10.6444464	9.51307168	9.33953682	9.47700334	11.0003701	9.08106867	10.57253	10.0888051	9.46685452	9.23831152	9.93747334	9.19180717	10.7255461	9.27042153
10.4604179	9.02864311	9.23821372	8.97007337	9.94982188	9.39067457	9.91377936	9.76324444	10.9663707	9.75564972	9.36166908	9.31529553	11.2755475	8.94576041	10.4976185	10.1953823	10.5058856	9.52681799	9.87310893	9.2525285	10.9997318	9.46002759
10.5044424	9.1282206	9.11841261	8.89710848	10.3555816	9.37200744	9.59568123	9.57280891	10.5252038	9.54916822	9.30488005	9.44527882	10.780544	8.98427447	10.2639859	9.96664645	9.94481567	9.35345194	9.8803517	9.18424389	10.5755679	9.31811451
10.4850491	9.00801434	9.07432935	8.9450805	10.0966647	9.22673042	9.69849809	9.65464227	10.6034797	9.38291333	9.30504584	9.46780962	10.9056667	8.95438487	10.3141028	10.0506654	10.037611	9.29814812	9.96607413	9.05078164	10.7955786	9.26505701
10.2942327	9.09606811	9.09866517	9.00843518	9.96828667	9.29505865	9.88249053	9.72236607	10.7902798	9.6401773	9.86095437	9.53262262	10.7316681	9.46264308	10.1306381	9.91716991	9.99095601	9.17856409	9.93202521	9.40096752	10.7885142	9.39997013
10.4477627	9.42987612	9.21206769	9.21337996	10.1304231	9.46142703	10.0372599	9.79964074	10.7149701	9.69767324	9.93033718	9.57902226	10.9860683	9.39559875	10.448335	10.2566661	9.88478679	9.60820553	10.1128129	9.45145465	10.9100396	9.49129835
9.9166961	9.48079919	9.49153541	9.43755491	9.98988707	9.50315156	9.68261954	9.735283	10.3064834	9.71981528	9.83644924	9.73056153	10.6080581	9.68347557	10.0968157	10.0736089	9.87246413	9.53491234	9.89829991	9.66745535	10.6426873	9.49110342
10.1378297	9.48031138	9.64856265	9.29610577	9.82564178	9.60411759	9.75532419	9.65244863	10.4608221	9.71085308	9.90637154	9.64359708	10.7469752	9.71540493	10.1639468	9.98182105	10.0412088	9.37435543	9.8171361	9.57819035	10.6991232	9.49764358
9.93785696	9.64043865	9.59966411	9.68457828	10.0097086	9.68764082	9.72613422	9.66539172	9.86657952	9.74364298	9.95450519	9.75687922	10.6095939	9.74669558	10.010183	10.0868853	9.89068775	9.68148201	9.90402942	9.69837031	10.3585527	9.53253699
10.3201634	9.08618171	9.21504361	8.94475241	9.91179591	9.37634356	9.87913242	9.7547212	10.7583041	9.78476745	9.58806364	9.53007626	11.0979278	9.11987694	10.3957481	10.0591091	9.79667839	9.63363675	9.97981715	9.23997802	10.9037299	9.496622
10.0821247	9.27183096	9.24122548	9.18352845	9.95726417	9.37990975	9.72255164	9.58355908	10.4170018	9.68157741	10.0891993	9.47293679	10.880811	9.53402611	9.94716876	9.84409874	9.91366135	9.43558877	9.48968538	9.43913623	10.7731021	9.22659996
9.99033387	9.25652038	9.26914584	9.27092021	9.84116552	9.40710272	9.68844153	9.71763435	10.7242408	9.52462413	9.60433054	9.43192294	11.2466623	9.36393402	10.1389974	9.98205189	9.67188999	9.46371443	9.8624394	9.50190885	11.1236631	9.33607979
10.3960617	9.28490587	9.33080246	9.3320586	9.96247921	9.56603524	9.82105082	9.8281752	10.5054268	9.6328986	10.0971532	9.5671459	10.9532249	9.66017173	10.295725	10.2404412	9.97546343	9.90189746	9.85164166	9.67428568	10.7454967	9.35320799
10.0740131	8.97346566	9.19371886	9.07773905	9.98249294	9.22269416	9.98786342	9.68542656	10.609975	9.80231701	9.39059876	9.67248691	10.5743304	8.86612256	10.1496313	9.73784003	9.73286629	9.30057856	10.0798806	9.43170713	10.7066296	9.46902177
10.3906621	8.96266705	8.89868479	8.82735407	9.87465238	9.23017406	9.77058838	9.65709411	10.6955026	9.49838346	9.13383737	9.50776533	10.8952279	8.83131874	10.343004	10.0400732	9.83787351	9.3393832	9.94957925	9.12416405	10.8134902	9.19471149
10.2344636	9.17058548	9.23416638	9.13620419	10.0371083	9.42284341	9.69162024	9.67783042	10.7310871	9.6523247	9.68894567	9.67366373	10.7340599	9.36843173	10.3537558	10.3493568	9.78644599	9.40675657	9.97818907	9.24931457	10.8254624	9.36647388
10.1376425	8.91962759	9.03300247	8.87761268	10.0249369	9.12576829	9.64171339	9.61166362	10.7179174	9.38844439	9.43701694	9.49472244	10.9564229	9.15913757	10.4189107	10.1026888	9.63684408	9.48361156	9.89368268	8.88303077	10.7996995	9.06086648
10.1049868	9.30980673	9.26619184	9.12773087	9.77110743	9.47149313	9.90211232	9.79031158	10.6387433	9.76899098	9.56785034	9.63889433	10.6859858	9.30008457	10.0610941	9.85979092	10.3142044	9.61731881	9.95816829	9.48583725	10.6052235	9.5500778
10.3193577	9.2927603	9.28463337	9.13651539	10.137544	9.41409335	9.74994556	9.59448156	10.5888507	9.91529175	10.2848785	9.57765391	11.019951	9.51243015	10.3669087	9.99471847	10.0828809	9.45458505	9.49593798	9.47612592	10.7899703	9.29958715
10.1077976	9.28750077	9.28427389	9.19311381	10.1300624	9.45063489	9.77739651	9.60865194	10.6102893	10.0159351	10.3261213	9.72597635	10.8731784	10.0066041	10.165023	9.96484735	10.3368821	9.58560061	9.89935992	9.8712995	10.7180251	9.68650253
10.4468758	9.26408694	9.236991	9.59103373	9.98270303	9.46737758	9.66575497	9.58230279	10.2837507	9.52244003	9.73826512	9.75393339	11.1276302	9.32707761	10.7267454	10.4005314	9.45153838	9.64320301	9.95335431	9.42909562	11.2367331	9.30314717
10.2809315	9.37977511	9.36189236	9.27144484	9.80451122	9.54198204	9.92344698	9.97683285	10.8420212	9.77969132	9.46841504	9.80511984	11.2990714	9.35067805	10.2210781	10.0843929	9.8827545	9.56695549	9.90190213	9.51222802	11.0609694	9.45212152
10.4757373	9.27748567	9.38553393	9.22667411	10.0611182	9.5114037	10.0254856	9.89069781	10.7705213	9.66970172	9.43803069	9.58166945	11.2512281	9.10664029	10.4556572	10.1592618	9.37232269	9.12184827	9.87939847	9.43727761	11.0661534	9.15125612
10.7002885	9.16851176	9.4452313	9.65839927	10.3169151	9.61870088	10.0061521	9.79595788	10.540957	9.32182996	9.81519278	9.59243884	11.134867	9.1802278	10.5293059	10.5455479	9.4755993	9.68681762	10.0436839	9.26522376	11.0567918	9.2472924
10.2959563	9.26510389	9.3722036	9.23906617	9.96200904	9.57050448	9.90566468	9.82411659	10.7744894	9.73290697	9.40705167	9.70684852	11.0551592	9.06522441	10.1765732	10.0867896	10.0201206	9.60756029	9.94450611	9.39644469	10.9827111	9.45908215
9.75689976	9.02665464	9.1772872	9.14022449	9.37187214	9.34596783	9.859225	9.76265819	10.7700099	9.53601574	9.25683719	9.34890383	10.9773317	9.16784873	9.86749016	10.0530904	9.78512397	9.12039407	9.65889105	9.03317056	11.0452025	9.19131007
9.88837516	9.22134624	9.22863507	9.36910456	9.5611014	9.48158606	10.0340464	10.0253801	10.8375065	9.52878114	9.53173911	9.44767339	10.8620161	9.31262837	10.139329	10.1788377	9.58404428	9.42588937	9.77962714	9.1861872	11.0519334	9.42497706
10.4211991	8.77977222	8.83559796	8.64521478	9.77802697	9.26925778	10.3401271	10.0256436	10.6418174	9.96075288	8.94500866	9.39397204	10.9329767	8.85667627	10.1530794	10.0280997	9.81176361	10.1362358	9.97711516	9.50701808	10.9931019	9.88985442
10.5267445	8.84647395	8.72090631	8.69786414	9.85045142	9.0678982	10.1642297	9.94304118	10.7659345	9.90549632	8.97317809	9.14100137	11.0053512	8.7834067	10.2824586	10.0628601	10.0011897	9.30573896	9.8255174	9.35698956	11.021273	9.52059548
10.2451735	8.83528133	8.86838859	8.95684338	9.72805192	9.2324915	10.1019359	9.86927502	10.6073668	9.74828246	9.37157314	9.25248129	11.0616625	8.82924034	10.2598512	10.1508197	9.65628702	9.90607908	9.6519237	9.04992263	10.8239118	9.2809751
10.3777827	8.85695581	8.74828544	8.55416349	9.94263872	9.10721651	9.93908487	9.79693346	10.6590031	9.64208855	8.84816925	9.29480888	11.0272145	8.5390626	10.1162145	9.95467212	9.77477359	9.53160882	9.71815596	9.02170058	10.7842812	9.50288055
10.4523936	8.8444046	8.8840241	9.08188825	10.1383884	9.37518511	10.1056939	9.82505451	10.6288124	9.69971386	9.64078651	9.61202863	10.8438626	9.17744453	10.5361817	10.3584026	9.93071373	9.8464021	9.71913043	9.17266553	10.7417345	9.43130996
10.3701121	8.61421177	8.77972335	8.59876438	10.0934398	9.16728052	9.8184647	9.78076581	10.6746474	9.57878056	9.09341724	9.37068773	11.1271839	8.52539747	10.1039535	10.3019422	9.87433938	9.88616394	9.52126637	8.75798459	10.9524	9.14642519
10.5821491	8.63962352	8.61093332	8.46136272	10.1393016	8.96559899	9.9604528	9.81898943	10.6931221	9.59734436	8.95217742	9.36055514	11.0542084	8.57629795	10.3707895	9.99954257	10.2751855	9.45743957	9.91752316	8.96649889	10.8520428	9.32779771

PTPN7	QKI	RAB8A	RALGPS2	RAP2A	RAPGEF2	RASGRP1	RBBP6	RGS5	RHOH	RPL4	RSAD2	SFRS2B	SFRS6	SIPA1L3	SLC15A2	SNTB2	SPARCL1	SPOCK1	SUPT16H	SYNC1	TARP
9.47223838	10.011869	10.0325658	8.97131999	10.8995693	10.4227051	9.93351346	10.3249625	10.4112596	9.84993667	10.837947	10.4968049	8.81937936	10.7534455	9.30900628	9.38092628	10.5976469	10.9353654	10.4528487	10.556278	10.0169743	10.1046932
9.80889034	9.97793979	9.83297559	9.58591424	11.4873149	10.5129977	9.71247373	10.2501965	10.6715087	10.1122791	10.8094458	9.93142748	9.63882851	10.8319886	10.1900377	9.78392357	10.7137936	11.5529349	10.5877868	10.4668338	9.77749248	9.82517484
9.35487498	10.3083202	9.70944922	9.10452628	11.2401359	10.5871344	9.12400992	10.0434656	11.2815973	9.74090003	10.9979887	11.0625515	8.94327271	11.474877	9.99599464	9.09750183	10.7884213	11.4051678	10.7340065	11.1891194	9.91154118	9.52852427
9.37519857	10.4380552	9.54096354	9.01947908	11.5204483	10.6344276	9.88451143	9.94205822	11.4393805	9.8369688	10.7828601	10.3002305	9.18073927	11.4589506	10.082365	9.46995778	11.245971	11.1002615	11.0793961	11.513194	10.5251136	9.54666001
10.005616	10.2449965	9.82204824	9.09443303	11.0802732	10.2578812	9.9912317	10.1140401	10.6001565	10.4541227	11.1437093	9.5229752	8.7751424	11.2105069	9.50327317	9.35892564	10.7570315	11.3598541	10.6246658	10.8902915	9.77081818	10.790635
9.63711821	10.231185	9.61640397	9.16693316	11.2585479	10.2265569	9.50842336	9.66785726	10.5105818	9.61039277	10.9656336	10.4890408	8.99869338	11.2962529	9.48782543	9.30039524	10.1869766	11.3492089	9.91625774	11.1195613	9.81412557	9.96389398
9.11492831	10.2085757	9.58779314	9.09911983	10.8135122	10.2097955	9.35823804	9.91839922	10.9545766	9.52925121	11.1851574	9.54575029	9.1422296	11.1676426	9.56633223	9.20598819	10.8713015	11.5172315	10.5385186	10.7535537	9.85746085	9.16549159
9.37451413	10.1060106	9.58680906	9.38642031	11.2931876	10.2538857	9.44042419	9.79090943	11.2637313	9.57865146	11.0432071	10.0572038	9.0041074	10.9741245	9.66335567	9.58990894	10.9720888	11.1264874	10.5873963	11.1014555	10.1083423	9.83768649
9.15235399	10.4111235	9.78878188	9.03408536	11.1402722	10.4370317	9.4386348	10.1340089	11.0918072	9.51305615	11.0849757	10.2913805	8.99637385	11.3732009	9.49731359	9.26867387	11.0207794	11.3668799	10.3355671	11.0504954	10.2715564	9.4521492
9.90950804	9.97871994	9.85331643	9.49673546	11.2873468	10.5864342	9.89411877	10.3594248	10.3574263	10.0841121	10.8062916	10.4484187	9.70788556	10.9250845	10.1143704	9.76318484	10.7248705	11.2576279	10.5897753	10.3437845	10.0456388	10.0078685
10.149976	10.0394187	9.78740333	9.12640274	11.2071436	10.204955	9.85719722	10.0040569	10.8042228	10.0626624	10.9659555	9.62422181	9.22900898	11.2811283	9.55346114	9.50055919	10.4670093	11.291752	10.3942343	11.0593732	9.62339	10.0097323
9.4510158	10.3521952	9.6959036	9.01828546	11.1304255	10.4410595	9.38312573	10.0167648	11.2523885	9.62020041	10.9716863	9.85167586	8.9184717	11.3521843	9.65235836	9.36664636	11.0029418	11.2446666	10.3437928	11.116225	10.1183415	9.50472465
9.40400829	10.4867655	9.85704797	9.07850035	11.0173205	10.6235309	9.35552521	10.1580029	11.2775975	9.63724421	10.7015485	10.1782856	9.11248772	11.3371695	9.80448551	9.47417722	11.1199127	10.9204087	10.6019502	11.1425706	10.4737174	10.3169917
9.61212822	9.68822123	9.57467698	9.46539257	11.5884439	10.3226658	9.51708184	10.0482618	10.3066169	9.6865292	10.7039876	9.77974429	9.49040498	11.0081663	9.76694321	9.49601445	10.6262783	11.4573653	10.5145294	10.0943893	9.67025608	9.6416759
10.196935	10.405956	9.68893835	9.29290557	10.8345481	10.2889767	9.68579317	10.1532166	10.6166224	10.0896542	11.305792	9.78519112	9.27290658	11.4183142	10.00942	9.51257272	10.5728107	11.4128261	10.3533606	10.9104557	10.2031763	10.3595934
9.71609506	10.0115828	9.30244854	9.17839424	10.8563717	10.5373977	9.84349399	10.4506266	10.8994529	9.80771045	11.1404857	9.4918353	9.18325677	11.3713859	9.78607342	9.42265777	10.5332759	11.4166194	10.70346	10.8418504	10.3165118	10.0259815
9.61940292	10.3777793	9.71613427	9.62908174	11.2513812	10.2701148	9.37146893	9.9188228	10.8975834	9.81299398	10.4920278	10.0142746	9.40945794	11.1429494	10.0570651	9.58161082	10.9233046	11.1637595	10.3432868	11.0955641	9.95305921	9.51564413
9.91091514	10.1042376	9.859781	9.16642783	11.3118177	10.6924514	9.42930404	10.3776428	10.7633762	10.0910029	11.1931871	10.3729562	9.91519735	11.127381	10.1704879	9.4245987	10.9314204	11.4037638	10.9036131	10.7808878	10.1052433	9.76468851
9.76043096	9.99045927	9.86548408	9.15597907	11.6161237	10.7066702	9.48354397	10.3358671	10.6408919	10.293959	11.3363522	10.7627072	9.76740252	11.267243	10.3661511	9.36770138	11.0045021	11.6314046	11.0295696	10.7906147	9.84683697	9.6832544
9.29918557	10.1171454	9.9686524	9.30710466	11.2080763	10.7520796	9.77318925	10.5019818	10.8178463	10.3781915	11.1598354	10.5176816	9.90651018	11.079255	10.5252445	9.56684567	10.9102405	11.272544	10.5141116	10.8848747	10.0774997	9.90475132
9.85648183	10.179731	9.70072373	9.15624516	11.5697783	10.4802542	9.59662686	10.2034315	10.603617	10.1538163	11.3139925	10.767585	9.40045313	11.1577393	10.0956475	9.43413647	10.9472217	11.6346225	10.9123283	10.7294342	10.1731218	9.97647743
9.89186057	10.1252342	9.8252684	8.97091032	11.5467907	10.8874176	9.38248706	10.5013675	11.0014532	10.1680846	11.4262946	10.088206	9.61660445	11.3082055	10.232682	9.13368158	11.0031929	11.7090123	10.8946188	10.9200571	10.1399006	9.61256728
9.62278488	9.7202728	9.4593001	9.02199841	11.0671788	10.4417859	9.3044786	10.2041554	10.4851738	9.85078286	10.9520438	10.3601751	9.58593141	10.8371667	9.99219061	9.1517298	10.6167834	11.2794404	10.507275	10.3776698	9.73799588	9.43841698
9.69636452	9.84048799	9.44243994	8.86065734	11.2305808	10.5211109	9.27022934	10.1975622	10.4003284	9.93079458	11.0773507	10.0081397	9.67287113	10.9576662	10.0588421	8.93885615	10.7676243	11.2906552	10.6611228	10.5070823	9.81265145	9.47253257
9.66148971	9.9383837	9.59939638	9.12017662	11.3528649	10.6809741	9.53744528	10.4452142	10.9207758	9.98249351	11.1429834	9.74772753	9.38709233	11.0186813	9.93217061	9.25863062	11.0841306	11.4311453	10.7154126	10.5111414	10.3255684	9.61109082
9.76820029	9.72027825	9.80185211	9.28212807	11.1867701	10.6851131	10.0014185	10.4942955	10.4717367	10.2014875	11.0318496	10.2051937	9.92216722	11.0328978	10.1982463	9.73558482	10.8305081	11.2451906	10.7273448	10.5992871	9.94317539	10.0296351
9.8168914	9.89800863	9.73868805	9.47967805	11.0143102	10.1870056	9.74434025	10.0904734	9.9575292	9.99312184	10.7594921	9.68649654	9.81899588	10.7824651	10.1279026	9.71378464	10.2233344	11.0196356	10.5935274	10.3260268	9.80105511	9.79569987
9.69025183	9.85469906	9.58543971	9.3547072	11.3134084	10.3371554	9.47070697	9.92096687	10.1920488	9.84982483	10.9468408	9.60707821	9.51389036	10.7398747	9.90102804	9.54983733	10.5254603	11.4043382	9.92236971	10.2577512	9.63150429	9.61731928
9.86797564	9.89402343	9.76998368	9.62656709	10.6937702	10.01435	9.76430754	9.82115873	9.81698901	10.047142	10.319627	9.72798069	9.70904137	10.3822427	10.0558187	9.77414493	9.86195416	10.4561238	9.73993984	9.96788785	9.69422431	9.83436965
9.81551531	9.94899469	9.77245978	8.9430298	11.2980511	10.6270384	9.73299492	10.4528042	10.7028708	10.0445102	11.1235123	10.0886774	9.4782872	10.997873	10.0918866	9.27557345	10.9227994	11.378193	10.5705218	10.5527367	9.93269868	9.85540277
9.44600528	9.66960208	9.70978822	9.29785475	11.2422079	10.4791131	9.39912928	10.081194	10.3871852	9.99051958	11.1757692	9.78742243	9.49052125	11.0281442	9.7736917	9.46765068	10.5809055	11.4652993	10.2611329	10.3423237	9.7304064	9.68291995
9.60380412	9.89353914	9.74354955	9.29736553	11.6646713	10.5416503	9.40617932	10.0514295	10.5209578	10.0263758	11.2814531	9.43944156	9.41009259	11.1344598	9.97238358	9.37267196	10.6426247	11.5162838	10.7759349	10.5909228	9.65918582	9.40237626
9.78174829	9.88520485	9.92143552	9.32908683	11.1816408	10.5325275	9.60910761	10.159713	10.268345	10.2094659	10.9353238	10.0581043	9.61728204	10.9049385	10.1565681	9.73979103	10.6166483	11.2199114	10.3967565	10.5769198	9.78531142	9.78781625
9.78669869	9.9078085	9.79209474	9.07799254	11.1270454	10.6847155	9.47614081	10.4974733	10.9824377	10.0626977	11.0465942	9.51755061	9.40488545	11.0163177	10.0995209	9.31572701	10.7679201	11.2142973	10.6592811	10.5887473	9.83096288	9.61818493
9.266096	9.52613634	9.76521418	9.01689954	11.0790401	10.5551363	9.31002665	10.4211225	10.7590694	10.0436828	11.2153109	10.1817767	9.20823346	11.1886471	9.97701979	9.13137107	10.9134991	11.4825133	10.6660436	10.6729787	9.64035307	9.51724881
9.91106095	9.90107768	9.83423911	9.24164787	11.2188271	10.4846988	9.57642603	10.3222888	10.2143709	10.0948996	11.0237562	9.96967892	9.58265754	10.9297602	10.1108931	9.43461787	10.6647991	11.1737218	10.4578439	10.6322354	9.80628662	9.9978714
9.77494895	9.82206586	9.5128877	8.95796137	11.3267277	10.5030315	9.2846321	10.250994	10.2076704	9.96200649	11.2779559	9.92662321	9.22646483	11.0572876	10.0995703	9.11920918	10.8106523	11.3293785	10.6162004	10.6990857	9.72773572	9.68908204
9.90128825	10.1839791	9.77429007	9.30333605	11.1468732	10.4607427	9.64544485	10.3782513	10.5938045	10.0950994	11.054451	9.99271722	9.71685599	10.9160507	10.0995618	9.55104527	10.8171693	11.3146238	10.7464423	10.6175623	10.5072461	9.82790177
9.7059695	10.0051302	9.85081711	9.19598663	11.4482234	10.4204952	9.34632398	10.0291568	10.3399122	9.93552876	11.0378189	9.59341959	9.21945402	10.9246911	9.626728	9.26039029	10.5375084	11.4416083	10.5028654	10.3884358	9.45759639	9.39006381
9.81452754	10.1156896	10.1096285	9.54384946	11.292297	10.8319007	9.73203098	10.4200428	10.7285736	10.433323	11.1168407	10.2158672	9.66739014	11.0267133	10.1617893	9.76849841	11.0948646	11.4568811	10.6774119	10.6650523	10.1094538	9.99489443
10.0327486	10.2345321	10.212465	9.37266138	11.8662572	10.4876793	10.03446	10.1599441	10.1500088	10.3589777	11.2842751	10.3502998	9.47575018	11.0828502	10.1194674	9.56722744	10.5494201	11.594581	9.49735879	10.816327	9.65747905	9.7210787
9.7787532	9.97752693	9.70877727	9.19800316	11.4886409	10.3544559	10.0002144	10.1941095	10.6803649	10.1362777	11.3301924	9.6713889	9.45488879	11.1199529	9.87772312	9.59668573	10.8704029	11.6559066	10.5334518	10.6874928	9.76994977	9.51216412
9.9043562	10.3013871	9.87746829	9.10415879	11.2149791	10.5210677	10.0349629	10.2543208	10.4337494	10.2399873	11.0625634	9.81697682	9.47125685	10.9655923	10.0528618	9.58491284	10.7287507	11.3457009	10.9097239	10.8806791	10.1415559	9.61816967
10.0094797	10.2025077	9.85329407	8.9788376	11.2657566	10.5887388	10.0048242	10.3821861	10.117561	10.2811078	11.2092108	10.1991741	9.58929986	11.0803197	10.2677417	9.53996405	10.469682	11.2266858	10.5919451	10.8455221	9.52316664	9.81372684
9.86676947	10.1469341	9.64757945	9.19262171	11.2752382	10.713048	10.0548277	10.4412352	10.7941972	10.1758928	11.0986947	9.86829579	9.62823141	11.0842275	10.310832	9.74500133	10.9671985	11.3119711	11.0569881	10.8221335	10.1624821	9.78642888
9.5313994	9.55575982	9.27564801	9.07866009	11.2734125	10.6678329	9.76695986	10.476365	10.4351261	9.79085202	11.2078893	9.36005737	9.28106172	11.2026358	9.86322768	9.46604508	10.8966884	11.465938	11.0376812	10.8508972	9.87413645	9.82393904
9.74410706	9.69885958	9.39209297	9.16629348	11.3711695	10.7746108	9.94135795	10.5122161	10.1429781	9.99037733	11.2051766	9.5095556	9.31435714	11.1216761	9.83470786	9.60369473	10.7406621	11.3224084	11.0058104	10.7731192	9.74334668	10.0388581
9.4145434	10.4467254	9.99151952	8.8168983	10.9554825	10.6946483	9.62914821	10.6385105	10.9003448	10.0371249	11.0888725	9.80029646	9.34728727	11.0001866	9.57498278	9.53432545	10.8814652	11.2514256	10.8647632	10.859328	10.3071185	9.49289757
9.19867854	10.2702536	9.95148296	8.62390087	11.1169771	10.7114927	9.42097779	10.5581729	10.9607052	9.89564802	11.1565481	10.2843596	9.43789268	11.0552034	9.60973729	9.26798316	11.0852802	11.3056053	10.7970542	10.7781042	10.3484916	9.52567219
9.60501824	10.0502987	10.0475899	8.63879728	10.9321581	10.3680592	9.61284853	10.3018018	10.570522	10.0589606	11.039464	9.461563	9.46690386	10.9070787	9.85806492	9.32677357	10.6652036	11.1495044	10.7880943	10.5977862	9.80665614	9.71297261
9.45842191	10.0776065	9.89257232	8.70522669	11.0188435	10.5724019	9.54438932	10.4088352	10.9139125	10.1338376	11.0177844	10.1677092	9.48936171	10.9300007	10.0566424	9.3434392	10.8996878	11.252365	10.8609079	10.5866163	10.1125788	9.56105755
9.50531128	9.9321722	10.0264403	8.61157247	10.8005477	10.480006	9.93807903	10.1488926	10.5586552	10.0418031	10.9156208	10.2223876	9.41265663	10.7946708	9.97094381	9.2242765	10.63042	11.0361521	9.99440579	10.5670978	9.96465531	10.0172999
9.38331033	9.75977961	9.46063928	8.48514355	11.138184	10.4549804	9.82078072	10.4011675	10.5753168	9.97946386	11.0770487	9.63990752	9.04781772	11.0613035	9.78921164	9.14996865	10.8362864	11.2444205	10.9576448	10.8068513	9.99814994	9.45654989
9.4266678	9.95430785	9.84387216	8.67221241	11.0534714	10.4895761	9.6389754	10.4470511	10.8352414	9.84563762	10.9390819	10.4324568	9.04787555	10.9831168	9.83923692	9.37153463	10.8430012	11.1798178	10.7373948	10.6465263	10.0740615	9.42241278

TBC1D20	TBC1D24	THRAP3	TLE2	TMEM43	TNFSF8	TOX	TSPAN3	TTC3	TUG1	UCKL1	WDR90	ZFHX3	ZNF839
10.0231159	9.50484662	10.7540628	8.88506591	10.7773398	10.5804778	9.96212253	10.9479356	10.4535725	10.5265443	9.41377589	9.4872502	8.87189442	9.14433378
9.88968628	9.80049068	11.0424038	10.3638952	10.9525381	9.72309502	9.70965597	11.4348809	10.4055331	10.453899	9.68529087	10.220679	9.65920621	9.89904311
10.3352823	9.14226985	11.6347512	9.79530138	11.1223796	10.1889538	9.34539388	11.1488958	10.630738	11.091935	9.34256146	10.0023995	9.02971877	9.64212468
10.2883398	9.47059103	11.5127842	9.75772375	11.1688738	10.0146929	9.49120802	11.087749	10.5821975	11.0818339	9.66725002	9.53007542	9.42431812	9.35678836
9.99110997	9.39656601	11.1891721	9.36189425	10.9271138	10.725795	10.064463	11.1956366	10.5899293	10.98156	9.2817328	9.37299716	8.98741123	9.37463439
9.89527993	9.35911475	10.9476415	9.49506201	10.7865263	10.7865586	9.44488014	11.0808634	10.2613024	10.8730144	9.21452342	9.35929919	9.22183693	9.36268806
9.9265974	9.48959493	10.6771078	9.49471069	11.1105855	9.91688962	9.34587295	11.1707682	10.4106531	10.9218491	9.61304943	9.63704214	9.09355893	9.45804574
10.018513	9.59918167	11.0896249	9.61237783	11.0918642	10.2414055	9.4609504	11.1049987	10.5884348	11.0624721	9.66288805	9.5701709	9.11630535	9.4115835
10.0994833	9.44785362	11.1480819	9.44174812	11.0684536	10.3067396	9.35747393	11.2162299	10.5982298	11.0108789	9.45206361	9.44988241	9.46725299	9.53378411
10.014349	9.91426975	11.1048638	10.2361332	10.7823784	9.69044636	10.0114465	11.241529	10.5034921	10.4836977	9.71815477	10.2786488	9.74944857	10.0194107
9.94019536	9.35903518	10.7994218	9.42243757	10.8190737	10.8420545	9.63111048	10.9989909	10.4949826	10.9939534	9.45275877	9.46809994	8.96479994	9.5218552
9.90602582	9.22983483	11.1502769	9.54998923	11.0184774	10.1633049	9.44581385	11.0185664	10.300427	10.9804407	9.56617907	9.57743755	9.1212542	9.45361734
10.2740997	9.09222097	11.2792158	9.71783647	10.9811876	10.3038874	9.41641338	10.6392762	10.6078295	10.9599096	10.0465533	9.88389692	9.32025267	9.83841696
9.70393756	9.4826769	11.1688603	9.88943753	10.6495948	9.47229823	9.5883558	11.4285235	10.2108053	10.1600009	9.63416728	9.67851149	9.46105341	9.64142393
10.0806484	9.48368584	10.9949295	9.96131467	11.1584104	11.0851803	9.72499521	11.1252339	10.5464007	11.0369189	9.81771324	9.85443554	9.31651109	9.72190918
10.1360701	9.50865349	11.0287688	9.7809709	11.1598457	10.5509888	9.53545324	11.1203146	10.68655	11.0279466	9.68093701	9.87846794	9.37445569	9.68025401
10.0172716	9.3530876	11.1246265	9.82365956	10.7654179	10.1120154	9.49274096	10.7606939	10.2949675	10.6818452	9.79935723	9.63134005	9.74604976	9.65197776
10.1937906	9.82095844	11.1435808	10.4176405	11.0477314	9.67500643	9.52343911	11.3442957	10.7256704	10.6220739	9.64628283	10.053889	9.55453842	10.0235034
10.0388666	9.45698736	11.2076093	10.475844	11.2055252	9.51950586	9.61998592	11.5982141	10.7298701	10.6016457	9.50378706	9.86265305	9.24718178	9.86404016
10.305269	9.57765447	11.096207	10.6597122	11.0595644	9.82553413	9.56090025	11.2712598	10.7311873	10.7555265	9.95681312	10.2748508	9.78606042	10.2475802
9.95496512	9.2127759	11.1882671	10.5644039	11.1287652	9.43880186	9.38712815	11.4695959	10.3780018	10.3283172	9.45430134	9.74691975	9.27922007	9.68602913
10.1266999	9.35174252	11.2469763	10.6431715	11.3418124	9.41268657	9.42103245	11.5614754	10.8072124	10.8468791	9.72640639	9.90214953	9.19492408	9.93355294
9.72437874	9.59794802	10.8273156	10.2369872	10.937041	9.23522188	9.27006284	11.2054571	10.4295619	10.322391	9.43696301	9.73892917	9.13501757	9.67998285
9.81949375	9.63148654	10.9350015	10.3324424	10.8378011	9.26375114	9.42055845	11.3157568	10.4123332	10.4529497	9.45032775	9.75455043	9.20706128	9.70179573
9.85811603	9.55605262	11.1424065	10.3668896	11.1207638	9.35469729	9.48636291	11.5170834	10.5896713	10.5665228	9.79973168	10.2070222	9.34178156	9.91194795
9.9957096	9.71819412	10.8398005	10.4488114	10.9804544	9.47608252	10.0812277	11.2259571	10.7734483	10.6293791	9.75467612	10.3504212	9.49876593	10.022453
9.89737688	9.70717867	10.6249044	10.2341616	10.6893075	9.66906257	9.70587259	11.1567502	10.4830977	10.2681481	9.84737308	10.1988796	9.65304793	9.96996122
9.65682062	9.54526654	10.7749642	9.99545504	10.8468403	9.37987937	9.54370373	11.2947376	9.97213336	9.96580689	9.69554039	9.90166675	9.34481048	9.61651005
9.82686596	9.73339029	9.98359661	10.2655492	10.201529	9.7430596	9.84440068	10.8836202	10.1815063	10.0190234	9.79064654	10.2475928	9.70785884	9.9088296
10.040009	9.33866077	10.9152908	10.298731	11.0066149	9.45999403	9.74204516	11.2549456	10.5969656	10.5302188	9.54314412	10.1634335	9.32301575	9.8997408
9.79953699	9.6655566	10.9828294	10.0776352	10.9229314	9.48924625	9.72304663	11.3527657	10.5368775	10.5080573	9.68960983	10.4389872	9.47736138	9.96472056
9.69424336	9.32392109	11.0031069	9.88284148	10.7531806	9.31980038	9.31465162	11.3839668	10.5892172	10.0458635	9.55999291	9.64339498	9.2965267	9.43374641
9.95219893	9.83961636	10.8853027	10.2227972	10.7050549	9.55100734	9.8166026	11.1541665	10.6863446	10.5090525	9.85650893	10.3499264	9.57550117	9.9330306
10.0078604	9.13643104	10.9539455	10.3842417	10.9543855	9.47243993	9.32829068	11.1626601	10.5297629	10.6072527	9.73704435	9.93652838	9.35732831	9.88812298
9.94632439	9.03300293	11.0379407	10.3545497	11.1357036	9.39375042	9.1139659	11.4305095	10.7209541	10.6430754	9.65751587	9.93538788	9.28849515	9.93647642
10.0177417	9.24276797	10.8436638	10.3212764	10.7530089	9.47571371	9.79732145	11.167383	10.633327	10.5093853	9.64039678	9.96067622	9.4377849	9.94870776
9.84215784	8.99179216	11.013964	10.165912	10.8838406	9.29166328	9.30633663	11.4420924	10.6950586	10.5654173	9.80915118	9.7066114	9.07768297	9.65677891
10.1546755	9.46346992	10.9897892	10.3693807	10.9493872	9.66895425	9.58543279	11.2771871	10.6927034	10.5826219	9.43474858	10.2484758	9.60638059	10.1642889
9.64962821	9.17618492	11.0637932	10.0126816	10.8399423	9.25069102	9.27651352	11.3634391	10.3809331	10.1126063	9.64981385	9.6239606	9.15962061	9.45468716
10.1808856	9.96757026	11.1227702	10.391595	11.1049927	9.80948168	9.94981777	11.4406767	10.7316451	10.7685555	9.55880286	10.8518396	9.86397038	10.2897619
9.65608791	9.31008254	10.9477951	10.3289523	10.7969472	9.40394669	9.31514841	11.3656235	9.99781955	10.2908854	9.44394491	9.56746331	9.27508611	9.57566222
9.80057511	9.38591326	10.8140472	10.0559242	11.124707	9.4059361	9.25412313	11.4645247	10.6145439	10.5294645	9.78149529	9.54990901	9.3242251	9.79106025
10.0410998	9.31037086	10.920408	10.4885599	11.1033287	9.54678547	9.35846516	11.3491958	10.9505596	10.6721711	9.11052669	9.87941239	9.33482044	9.9652856
9.86781194	9.05476404	10.7629697	10.2515346	10.7262015	9.25192633	9.30151547	11.0948121	10.53716	10.4770509	9.58996299	9.78449005	9.12043138	9.62160507
10.0960305	9.48933415	10.8286167	10.4648158	11.0065417	9.56934532	9.53751879	11.2547924	10.9966381	10.7583447	9.65932126	10.069391	9.53947727	10.108414
9.88021609	9.30342763	10.3720612	10.2532728	11.0596696	9.31555443	9.25464697	11.3970068	11.1581113	10.7879567	9.44450759	9.66620068	9.2172977	9.69964561
9.81210571	9.3768064	10.3417396	10.0031639	10.9737219	9.33796375	9.55975099	11.2715868	10.8885081	10.6764602	9.48555138	9.75917254	9.30347003	9.65867605
10.3207546	9.21141246	10.9256577	10.1703	11.1129526	9.35789514	9.41847532	11.2245019	10.7688843	10.7613973	9.91874744	9.9170326	9.21455949	10.1689794
10.2687639	9.32137771	10.9685291	10.1052429	11.1745312	9.14462959	9.38232629	11.3215247	10.8390468	10.6302421	9.71791076	9.7310513	8.95114678	10.0630419
10.1653393	9.07426197	10.7766975	10.0338623	10.9206275	9.37640929	9.43979769	11.1315042	10.7537758	10.5912647	9.39874197	9.69077338	9.01917157	9.88584632
10.1592292	9.20277722	10.830106	10.0679899	10.9735744	9.2121051	9.21981031	11.2728885	10.8346929	10.4707172	9.51046247	9.81245428	8.80152856	9.76517553
9.95814329	8.79517652	10.7724857	10.0822734	10.8403189	9.45543024	9.72349431	11.0347234	10.4442117	10.4908538	9.56481213	9.72248948	9.11714012	9.91039767
10.200658	8.79471697	10.9088345	10.083352	11.0408081	9.13874645	9.26041785	11.263143	10.8787658	10.7830826	9.70406997	9.57321854	8.76416216	9.79617782
10.1152325	8.97531778	10.9063838	10.0403875	10.989907	9.25310585	9.30115817	11.1674519	10.6780249	10.7008459	9.36791573	9.63614556	9.17867929	9.95264856
