# Supplementary material for: Heterogeneity of Synovial Molecular Patterns in Patients with Arthritis
Source: PLoS One. 2015 Apr 30;10(4):e0122104. doi: 10.1371/journal.pone.0122104 (PMC4415786; doi:10.1371/journal.pone.0122104)
Supplement: S2 Table — Raw Ct values from 31 UA samples are displayed for all the probes present in the Taqman low-density array. (PDF) [file pone.0122104.s003.pdf]

ID	ARL1.Hs0102	ARPC4.Hs008	ASH1L.Hs002	BAG2.Hs0018	BCL11B.Hs00	BSG.Hs00942
DIA060	24.81	22.55	24	27.24	30.11	24.25
DIA084	24.35	21.97	24.27	27.29	27.8	23.32
DIA091	24.37	21.76	24.04	27.37	28.58	23.65
DIA096	24.92	23.08	24.51	27.46	30.84	24.63
DIA106	23.72	21.93	23.71	27.69	28.21	22.99
DIA126	25.67	22.84	25.16	28.91	29.65	24.2
DIA133	25.21	22.97	24.21	27.98	30.75	23.44
DIA134	24.42	22.61	23.82	27.43	33.06	25.99
DIA136	23.83	21.62	23.33	27.66	28.45	23.13
DIA139	24.94	22.24	24.55	28.09	28.88	24.01
DIA156	24.93	22.46	24.41	27.81	30.38	23.62
DIA180	24.88	22.65	24.52	27.89	29.65	24.66
DIA190	24.65	22.32	24.41	27.9	28.81	23.53
DIA196	27.84	24.31	26.79	29.94	31.45	27.19
DIA202	24.74	22.25	24.01	27.51	29.45	23.22
DIA215	23.91	22.18	24.14	27.16	29.2	22.98
DIA231	24.69	22.47	23.36	27.37	29.89	23.34
DIA236	25.21	23.52	25.51	28.02	30.52	25.47
DIA289	24.29	22.11	23.81	27.39	29.8	26.09
DIA292	24.29	21.69	23.81	28.03	27.65	25.36
DIA297	24.78	22.67	24.2	27.36	30.5	24.2
DIA299	24.74	22.47	24.56	28.02	30.47	23.5
DIA306	24.95	22.92	24.62	27.84	32.04	23.98
DIA309	24.73	22.26	24.6	27.98	29.73	23.96
DIA321	24.92	22.86	23.84	28.18	32.5	24.76
DIA325	24.79	22.79	24.23	27.66	31.34	23.58
DIA348	24	22.26	24.28	27.89	30.11	23.84
DIA349	24.41	22.48	23.91	28.15	31.95	23.06
DIA354	24.86	22.57	24.52	28.19	29.25	26.12
DIA356	24.99	22.65	24.55	28.08	31.42	23.87
DIA358	24.74	22.61	24.23	27.54	31.71	25.68

BST2.Hs0017	BTBD1.Hs002	CALU.Hs0015	CCL5.Hs0017	CCND1.Hs00	CD209.Hs015	CD79A.Hs009
23.79	25	23.61	25.42	24.16	26	27.74
22.59	24.2	22.21	23.21	22.68	23.96	24.91
22.53	23.97	22.61	23.47	23.43	25.82	24.97
24.78	24.86	22.99	26.11	23.63	25.43	28.1
22.29	23.96	22.53	23.71	23.18	24.47	24.48
23.5	25.33	23.35	24.18	23.96	25.86	24.07
24.46	25.09	23.95	25.72	22.55	25.47	28.62
24.38	24.51	23.38	27.41	22.97	26.13	30.64
22.71	23.74	22.44	23.81	22.81	23.12	24.1
23.13	24.56	22.93	24.27	24.08	25.96	24.95
24.24	24.97	22.92	26.03	24.14	25.34	31.13
23.5	24.77	23.14	24.71	23.97	25.07	26.55
23.31	24.67	22.97	23.75	23.77	25.57	22.79
25.26	28.23	24.46	25.67	26.1	27.96	24.83
23.67	24.69	23.18	24.23	23.43	25.45	25.54
22.48	23.93	22.48	24.6	23.16	23.88	26.16
24.02	24.47	23.49	26.2	24.39	26.91	24.46
23.78	22.18	23.36	24.45	22.89	25.99	25.73
23.5	23.9	22.77	25	22.45	26.02	26.25
22.12	23.82	22.25	22.97	23.04	24.18	26.26
24.34	24.82	22.72	26.15	23.75	26.17	27.52
23.85	24.96	23.04	26.07	23.43	26.85	25.95
24.3	25.02	22.97	27.33	23.69	26.51	29.25
23.1	24.47	22.57	23.79	23.66	24.14	25.75
24.89	24.77	23.64	28.35	24.04	25.76	30.72
24	24.37	23.89	26.72	24.68	26.16	27.75
23.87	24.51	22.51	25.35	22.82	25.19	28.24
23.47	24.55	22.92	26.51	23.28	26.46	30.24
23.37	24.88	23.16	24.35	24.16	25.73	25.25
24.07	25.29	22.63	26.59	23.8	25.92	29.92
23.2	23.01	22.96	26.55	23.27	25.39	30.89

CDC42BPA.H	CDC42SE1.Hs	CHD1.Hs001!	CHD9.Hs003.	CHSY3.Hs005	COMMD2.Hs	CPSF1.Hs002
24.4	24.82	25.59	25.23	28.26	25.94	26.73
25.03	23.61	25.26	25.46	27.22	25.99	25.6
25.06	23.67	25.84	24.97	28.73	25.68	25.12
24.94	24.85	26.24	25.53	27.11	26.27	26.03
24.5	23.78	25.42	24.9	27.84	25.69	25.09
26.02	25.01	26.58	26.49	28.77	26.48	26.89
24.72	24.63	26.08	25.24	27.18	26.22	26.27
24.21	24.6	25.87	25.74	27.91	25.93	25.43
24.28	23.29	24.74	24.63	27	25.2	25.43
24.37	24.09	26.01	25.71	29.12	26.26	26.22
24.67	24.21	25.99	25.29	28.1	26.06	25.33
25.45	24.42	25.84	25.73	28.45	26.23	26.58
25.47	24.25	26.19	25.31	27.91	25.97	25.69
28.75	27.1	27.95	28.7	30.19	28.49	27.32
24.88	24.17	26.1	24.86	27.92	25.93	25.12
24.6	23.66	25.74	24.96	27.49	25.52	25.44
24.3	24.1	25.33	24.05	27.52	25.98	25.38
26.31	25.52	26.72	26.19	27.76	26.94	26.88
24.51	23.94	25.46	24.55	27.14	25.5	25.14
24.84	23.53	24.96	24.97	27.96	25.43	25.29
24.4	24.81	25.95	25.3	27.42	26.09	25.4
25.28	24.88	26.68	25.22	28.86	26.55	25.55
25.05	24.97	26.66	25.47	27.11	26.28	26.31
25.16	24.44	25.88	25.76	28.01	26.12	25.97
24.96	24.85	25.62	25.23	28.49	26.19	26.12
25.12	24.76	26.28	25.2	27.57	25.83	25.59
25.07	23.93	25.97	25.26	27.9	25.98	26.25
24.38	24.32	26.12	24.8	27.49	26.06	25.08
25.56	24.06	25.97	25.59	28.09	26.28	26.21
24.99	24.95	26.34	25.46	27.64	26.2	26.08
24.72	24.31	26.37	25.1	27.47	26.19	25.2

CST7.Hs0017	CTDSPL.Hs00	DUT.Hs0098	EIF2A.Hs002	EIF3L.Hs010	ERRFI1.Hs00	ETV6.Hs0023
27.72	25.89	22.55	24.04	23.48	22.47	24.72
25.77	26.39	22.88	23.8	23.42	22.05	23.96
26.96	25.62	22.43	24.88	23	21.75	24.36
27.56	26.37	23.13	24.52	24.16	22.62	25.29
26.5	25.35	22.59	23.6	22.91	20.89	23.6
26.23	27.55	23.55	25.02	24.56	23.9	24.53
28.52	25.78	22.69	24.69	23.41	25.05	25.06
30.24	25.84	22.46	23.91	23.07	21.42	25.36
25.71	25.17	22.24	23.59	22.35	21.21	23.55
26.42	26.58	22.86	24.68	23.79	22.69	24.46
27.67	25.78	22.99	24.3	23.6	22.83	24.85
26.67	26.24	22.98	24.64	24.03	22.54	24.73
26.03	26.45	23.25	24.49	23.65	22.9	24.62
28.99	29.63	24.62	26.82	25.89	27.29	26.39
26.59	25.9	22.84	24.36	23.5	22.28	24.42
26.81	25.67	22.61	23.92	23.01	21.17	24.15
29.01	25.72	22.12	23.95	23	22.74	24.51
27.81	26.93	23.9	24.83	24.22	25.86	25.57
26.72	25.62	22.83	23.99	22.31	22	24.78
25.42	26.33	22.26	23.73	23.23	22.9	23.45
28.57	26.18	22.98	24.44	23.62	22.8	25.18
27.91	26.31	23.13	24.64	23.61	21.97	25.34
29.38	26.22	23.18	25.02	23.68	22.7	25.69
26.01	26.86	22.96	24.29	23.44	22.88	24.62
30.45	25.2	22.77	24.22	23.46	22.14	25.66
29.01	25.49	22.35	24.34	23.49	24.14	25.63
27.56	25.86	22.66	24.17	22.75	21.81	24.79
28.96	25.47	22.58	24.49	22.73	22.67	25.17
26.81	26.7	22.75	24.46	23.66	23.71	24.58
28.23	26.14	22.77	24.68	23.11	23.55	25.05
28.46	25.48	22.78	24.14	22.79	22.12	25.13

FAM190B.Hs	FAM20A.Hs0	FKBP7.Hs005	FN1.Hs00365	FNIP2.Hs003	FRMD4A.Hs0	G3BP1.Hs004
25.86	24.58	25.18	17.24	25.56	24.75	23.41
25.25	22.93	25.13	15.87	24.72	24.3	22.23
25.64	23.51	25.18	14.73	25.41	24.46	22.32
25.8	24.45	25.48	16.64	25.79	24.95	23.37
25.74	23.17	24.72	14.77	25.23	24.19	22.91
26.85	23.73	25.8	16.89	26.05	25.55	23.6
25.21	25.38	25.72	18.83	25.33	23.95	23.21
25.03	25	25.08	16.61	25.07	23.94	22.97
24.44	22.85	24.38	16.12	24.39	23.32	22.07
26.61	23.69	26.09	15.78	25.83	24.81	22.92
25.92	24.88	25.66	16.92	25.69	24.94	23.45
26.09	24.14	25.28	16.87	25.74	24.79	23.28
26.26	24.15	24.93	16	25.68	25.12	23.07
28.22	25.93	27.05	18.98	28.45	28.91	25.23
25.99	24.66	25.07	15.62	25.55	24.49	23.16
25.64	22.77	24.69	14.89	25.49	24.06	22.4
25.56	24.55	24.66	16.21	24.9	23.97	22.87
26.81	24.72	25.61	18.65	26.44	25.99	23.83
24.8	23.8	24.54	15.3	24.59	24.35	22.84
26.16	22.28	24.83	15.34	25.41	24.37	22.36
25.16	24.43	25.19	16.82	25.32	24.44	23.2
26.01	24.12	24.43	15.38	25.12	24.8	23.47
25.4	23.9	24.21	16.47	25.88	25.23	23.6
25.67	22.64	24.98	16.18	26.09	24.73	22.71
25.45	26.8	25.51	17.67	25.21	23.87	23.04
24.81	25.97	25.7	18.39	25.26	23.68	23.29
25.58	23.4	24.42	15.65	24.99	24.82	23.02
24.78	24.67	24.75	16.4	24.87	23.83	23.03
25.69	24.36	25.99	16.85	25.49	24.71	22.98
25.35	24.04	24.52	17.27	25.55	24.94	23.57
25.26	24.07	24.81	17.12	25.48	24.66	23.34

GABRB2.Hs0(	GGNBP2.Hs0	GPR4.Hs009⁄	GYG1.Hs003(	HMGB1.Hs01	IFI27.Hs0108	IFI6.Hs00242
28.16	25.24	30.28	34.9	25.12	24.03	26.87
27.29	25.39	29.75	34.21	24.51	22.25	26.07
27.74	25.31	30.83	33.45	24.79	21.6	24.19
28.34	25.67	30.23	34.72	25.25	24.15	26.94
27.02	24.67	30.71	35.73	24.66	21.4	24.42
29.63	26.25	31.74	33.88	25.82	23.49	26.26
28.31	25.74	30.45	33.49	25.1	23.95	26.91
26.38	25.1	30.7	34.86	24.9	24.13	27.46
26.96	24.48	29.37	33.19	24.57	22.34	25.87
27.44	25.43	30.89	33.71	24.91	22.39	25.35
28.01	25.47	30.41	35	25.34	23.14	25.04
27.74	25.49	31.14	34.91	25.24	22.36	25.43
29.49	25.78	30.48	NA	25.3	22.81	25.54
28.26	28.52	33.59	35.33	26.59	26.36	29.59
27.36	25.3	30.08	33.88	25.01	22.71	25.87
26.75	25.51	29.21	33.06	24.87	22.26	26.13
26.26	24.87	29.98	36.25	24.79	23.87	28.01
29.07	26.62	33.33	34.47	25.33	23.67	27.92
26.35	25.06	30.97	36.01	25.4	23.23	25.6
28.8	24.91	30.57	33.53	25.16	21.98	25.63
27.91	25.72	30.94	NA	25.51	24.39	26.97
27.64	25.83	30.75	34.99	25.85	23.98	26.55
28.49	26.4	30.64	34.85	26.24	23.8	26.7
27.77	26.16	30.64	35.7	25.19	23.49	26.88
26.13	25.07	30.71	35.12	24.98	24.74	28.41
27.95	24.89	30.98	35.9	25.32	23.94	26.75
26.71	25.02	29.65	33.46	25.12	24.05	26.4
28.46	25.38	29.39	33.88	25.34	23.28	25.56
28.78	25.62	31.18	34.24	25.32	22.99	25.8
28.27	26	30.54	32.96	25.47	24.15	26.51
27.41	25.37	30.14	35.14	25.54	22.83	25.13

IFIT3.Hs0192	IKZF3.Hs002	IL7R.Hs0090	JAK3.Hs0016	KIAA0090.Hs	KIAA1377.Hs	LY9.Hs03004
25.96	28.47	27.34	27.48	25.98	25.23	29.83
25.32	26.56	25.12	24.45	25.52	26.83	27.43
24.31	26.38	24.87	25.05	25.28	26.64	27.11
26.17	28.72	27.77	26.73	26.18	26.25	29.36
24.21	26.3	24.78	24.43	25.19	25.92	27.71
26.47	26.22	25.22	25.62	26.3	27.47	28.06
25.66	29.6	26.62	26.72	25.97	26.47	30.2
25.55	30.69	29.61	27.45	25.72	25.57	31.68
24.7	26.53	24.55	24.25	24.7	25.94	27.77
25.72	26.38	24.93	24.99	25.67	27.29	27.99
25.82	29.39	26.53	27.13	25.82	25.68	30.29
25.58	27.51	25.91	25.48	26.14	26.53	29.02
25.67	26.06	26.1	24.84	25.76	26.69	27.24
27.55	27.77	28.21	27.93	28.35	29.44	29.81
25.7	27.6	27.15	25.83	25.58	25.59	28.4
25.57	27.25	24.95	24.56	25.34	26.89	28.15
26.43	26.97	25.32	26.42	25.67	25.32	28.95
26.48	26.78	26.9	27.32	27.14	27.68	28.33
25.85	27.42	26.62	26.22	25.09	26.02	28.57
25.05	25.83	23.19	24.02	25.27	26.09	27.2
26.83	28.45	27.21	27.16	25.98	25.74	30.06
26.5	28.27	26.6	26.77	25.88	26.84	29.63
26.97	29.48	27.89	27.94	26.03	26.58	30.71
26.5	26.87	25	25.92	25.64	27.16	28.07
26.98	30.96	28.17	29.1	25.9	26.16	32.68
26.21	30.09	27.99	28.57	25.69	26.26	31.1
26.96	28.5	25.9	25.89	25.44	26.4	29.31
25.93	29.78	28.3	28.06	25.31	25.96	30.6
25.56	26.61	24.94	25.21	26	26.95	28.32
26.91	29.36	28	26.84	25.98	26.51	30.09
25.45	29.96	28.34	26.65	25.63	26.21	31.25

MRPL52.Hs0	NAA25.Hs00	NBL1.C1orf1	NELL2.Hs001	OAS1.Hs009	PARP12.Hs01	PDE5A.Hs00
25.64	25.72	22.63	31.93	27.62	25.88	25.17
25.12	26.4	22.98	28.57	26.36	24.53	25.7
25.47	25.98	22.38	29.69	24.44	23.87	25.91
26.51	26.78	22.19	31.14	26.78	26.16	25.71
25.68	25.54	22.08	28.72	25.86	24.43	25.12
25.98	27.09	23.37	30.36	26.07	25.64	26.72
26.32	26.59	23.8	31.07	26.73	25.68	25.12
25.85	25.96	22.73	32.22	26.8	25.82	25.71
25.63	25.11	22.07	29.88	25.42	24.6	24.73
25.66	26.18	22.64	29.87	25.96	24.8	27.29
26.25	26.29	22.05	30.32	25.61	24.99	25.64
26.23	26.51	22.7	31.38	26.19	24.8	26.08
25.75	26.45	22.45	29.9	25.76	24.93	26.39
26.73	29.66	25	32.31	28.99	27.8	28.02
25.7	26.24	21.87	30.58	25.7	25.08	25.85
25.98	26.01	22.47	30.52	25.56	24.65	25.72
25.49	25.49	23.17	31.55	26.98	25.51	25.73
26.22	27.59	25.21	30.4	24.83	26.46	25.94
26.2	25.68	22.08	30.09	25.75	25.39	24.63
25.27	25.61	23.38	28.39	25.13	24.06	26.47
25.27	26.47	22.41	31	26.94	26.31	25.02
26.5	26.68	22.01	30.89	26.51	25.78	26.95
26.44	26.56	22.37	32.33	26.69	26.04	25.79
25.82	26.4	22.99	29.97	26.68	25.56	26.73
27.07	25.98	23.47	31.63	28.23	26.57	26.53
25.88	26.55	22.98	31.83	27.19	26.11	26.1
25.79	26.31	21.05	30.75	26.73	25.57	26.5
25.6	26.45	21.67	31.58	26.25	25.8	25.66
25.63	26.56	23.25	30.2	25.76	24.94	25.94
25.77	26.87	22.25	32.59	27.09	26.2	25.69
25.95	26.57	22.18	30.9	26.41	25.65	25.14

PGF.Hs00182	PHF21A.Hs01	PIK3C2A.Hs0	PTBP1.Hs002	PTEN.Hs0262	PTPN7.Hs001	QKI.Hs00287
25.91	26.14	25.5	26.64	25.05	31.57	23.5
25.24	26.11	25.88	26.33	25.24	29.01	23.57
25.08	25.59	25.52	26.04	24.38	28.76	22.99
26.84	26.07	25.75	27.13	24.85	31.56	23.75
25.18	25.11	25.15	25.92	24.29	29.09	22.85
26.14	26.6	26.78	27.21	25.93	29.03	24.18
28.11	26.09	25.15	26.71	23.97	31.47	22.92
27.76	25.38	25.16	26.54	23.85	32.63	23.53
25.22	24.68	24.45	25.33	23.63	28.87	21.98
25.23	25.93	25.89	26.56	25.3	29.24	23.1
27.12	26.09	25.68	26.84	24.72	30.81	23.23
27.17	26.02	25.81	26.97	24.78	30.12	23.24
25.84	25.97	25.63	26.48	24.81	28.6	23.24
27.58	29.14	29.29	29.43	27.95	30.18	26
25.82	25.71	25.3	26.31	24.58	29.74	22.71
24.71	25.14	24.6	26.22	24.26	29.21	22.86
27.03	25.21	24.72	25.84	23.79	30.79	22.84
27.9	26.67	26.82	27.69	26.16	30.32	24.71
26.14	25.88	24.74	26.66	24.11	29.99	23.18
24.8	24.88	25.32	26.05	24.18	28.58	22.57
26.89	26.1	25.51	27.1	24.51	31.2	23.46
27.12	26.25	25.91	26.86	25.07	30.47	22.68
26.12	26.25	25.85	27.09	24.69	31.76	23.63
25.51	26.41	25.87	27.22	25.38	29.02	23.14
25.81	25.94	25.07	26.52	23.33	32.74	22.3
27.84	25.71	25.61	26.29	24.43	32	22.91
26.03	25.91	25.54	26.47	24.45	31.09	23.2
26.14	25.9	25.45	26.65	24.08	32.06	22.84
26.06	25.99	26.03	26.76	24.64	29.59	23.3
26.09	26.4	26.12	26.8	25.47	31.34	23.2
26.4	26.3	25.35	26.36	24.37	31.94	23.54

RAB8A.Hs00:	RALGPS2.Hs(	RAP2A.Hs00:	RAPGEF2.Hs(	RASGRP1.Hs(	RBBP6.Hs00!	RBL2.Hs0018
24.35	27.85	25.17	24.89	28.9	26.84	25.1
23.1	26.8	25.29	24.32	26.55	26.21	24.94
23.24	26.62	24.24	24.84	26.54	26.14	24.68
24.38	27.61	25.09	25.38	28.67	26.69	25.48
23.22	26.1	24.65	24.57	26.19	26.09	24.38
24.07	25.85	25.73	25.89	27.12	27.66	25.68
24.44	28.2	25.01	24.79	28.81	26.94	24.91
24.12	27.92	25	25.03	30.54	26.43	24.73
23.22	25.77	24.54	24.46	26.21	25.58	24.04
23.57	26.72	25.18	25.79	26.78	27.14	25.35
23.97	28.37	24.95	25.38	28.83	27.08	24.99
23.98	27.45	25.08	25.64	27.45	27.32	25.06
23.63	25.59	25.29	25.25	26.79	26.83	24.97
25.15	27	26.92	27.44	29.28	29.66	28.85
23.94	27.19	24.81	25.03	26.86	26.84	24.72
23.49	26.93	24.2	24.57	26.95	26.64	24.5
23.96	26.64	24.61	24.73	28.22	26.41	24.38
24.17	26.41	25.42	26.16	27.65	28.02	25.96
23.61	27.32	24.75	24.23	27.87	26.61	24.8
22.56	26.81	24.12	24.66	25.75	26.35	24.12
24.18	27.13	24.97	25.43	29.69	27.41	25.02
24.52	27.97	25.56	25.38	28.67	27.2	25.3
24.61	28.11	25.1	25.31	29.85	27.51	25.36
23.65	26.73	24.87	25.58	27.43	27.41	25.14
24.59	28.25	24.99	24.93	29.83	26.52	24.35
24.73	28.58	25.09	24.82	29.69	26.76	24.55
23.99	28.35	25.12	25.47	28.09	26.92	24.89
24.13	28.25	25.11	24.96	30.3	26.94	24.56
23.55	26.68	25.1	24.89	27.19	26.82	24.95
24.33	27.61	25.5	25.45	29.39	27.32	25.9
24.06	28.23	25.27	24.98	29.17	26.97	24.95

RGS5.Hs0155	RHOH.Hs018	RSAD2.Hs003	SIPA1L3.Hs0(	SLC15A2.Hs0	SNTB2.Hs001	SOX4.Hs0026
22.1	29.53	26.69	26.43	30.64	26	34.17
22.27	27.83	25.8	25.69	28.8	26.6	35.39
23.23	27.36	24.95	25.66	28.24	26.22	32.6
22.77	30.61	26.77	26.12	30.09	26.19	35.02
22.39	27.04	25.11	25.4	30.39	25.62	33.72
23.79	27.89	27.27	26.42	29.13	27.01	34.57
22.52	28.08	26.65	26.22	29.4	25.47	33.71
21.9	28.12	26.74	25.7	29.46	25.64	33.47
21.75	27.18	26.16	25.16	27.71	25.06	34.93
24.05	27.82	26.76	26.02	28.62	26.86	35.48
23.52	29.28	26.01	25.99	29.61	25.73	34.73
23.4	28.66	25.45	26.1	29.5	26.15	35.46
23.62	27.2	26.53	25.89	27.38	26.18	33.46
27.17	28.06	29.25	28.34	29.91	29.48	33.81
22.75	28.33	26.55	25.7	28.37	25.85	33.73
22.75	27.89	26.74	25.66	28.25	25.97	34.67
21.38	28.85	28.14	25.48	28.26	25.79	34.78
24.23	27.91	27.39	27.07	29.94	27.54	27.12
21.37	28.72	26.48	25.66	29.03	25.84	32.6
23.35	26.93	25.44	25.62	28.13	26.48	33
22.86	30.19	27.88	26.21	28.92	25.99	34.31
23.85	29.05	27.77	26.55	29.48	26.22	34.17
22.53	30.75	28.25	26.53	28.71	26.03	34.32
24.17	28.89	28.03	26.56	28.83	27.44	34.8
22.35	31.86	28.34	26.55	29.67	26.51	36.17
22.5	30.98	27.29	26.38	29.4	25	34.74
23.1	29.57	28.51	26.06	28.86	26.08	33.86
21.89	30.83	26.46	25.96	29.35	25.27	33.84
23.25	28.13	26.25	26.1	28.63	26.66	34
22.84	31.34	27.53	26.17	29.75	26.31	34.34
22.52	30.17	26.15	26.35	29.59	25.75	30.65

SPARCL1.Hs0	SRSF6.Hs007	SUPT16H.Hs(	SYNC.Hs0025	TAF1D.Hs002	TARP.TRGC2.	TBC1D20.Hs(
20.97	23.73	24.69	27.37	24.95	30.32	24.84
21.79	22.55	24.46	27.18	24.41	29.98	24.3
21.06	23.14	24.64	27.27	24.3	27.8	24.28
21.41	23.68	25.16	27.72	24.99	30	24.97
20.48	23.22	24.38	26.92	24.41	28.06	24.13
23.38	23.81	25.71	28.85	25.74	29.25	25.62
21.66	23.84	25.09	27.77	25.31	30.39	24.69
20.88	23.4	24.65	27.52	24.55	28.11	24.51
21.08	22.34	24.09	26.54	23.94	28.21	23.92
22.93	23.24	24.99	27.93	24.83	30.22	24.85
21.22	23.68	25.06	27.64	25.01	31.62	24.97
21.89	23.57	25.12	27.62	24.92	30.29	25.01
22.07	23.48	25.09	28.11	25.06	27.88	24.51
25.95	25.95	27.64	30.97	27.92	31.69	27.07
21.18	23.44	24.93	27.06	25.14	28.45	24.23
21.16	22.76	24.48	27.5	24.31	28.94	24.3
20.85	23.31	24.68	27.48	24.22	30.23	24.04
23.1	24.55	25.59	28.92	25.99	26.75	25.64
20.66	22.79	24.32	26.91	24.74	27.18	23.67
22.44	23.01	24.07	27.64	23.93	27.73	24.58
21.61	23.62	24.96	27.45	24.84	31.14	24.97
22.03	23.75	25.24	27.78	25.4	31.34	25.04
21.86	23.79	25.54	28.89	25.28	31.97	25.1
22.97	23.2	24.94	28.79	24.94	27.91	25.07
21.28	23.64	24.62	27.3	24.44	32.94	24.32
22.11	23.75	24.75	27.08	25.22	30.81	24.2
21.07	23.22	24.74	27.98	24.83	30.3	24.43
20.84	23.68	24.83	27.24	24.94	30.68	24.36
22.57	23.58	24.96	27.77	24.93	26.6	24.87
21.52	23.52	25.19	27.31	25.67	30.88	24.89
19.67	23.5	24.67	27.05	25.19	27.58	24.51

THRAP3.Hs0(	TLE2.Hs0061	TMEM43.Hs(	TNFSF8.Hs00	TOX.Hs0020	TSPAN3.Hs0(	TTC3.Hs0159
23.62	26.36	24.95	28.19	29.78	22.52	23.9
23.6	25.71	25.26	26.62	27.05	22.7	24.02
23.71	26.95	24.94	26.6	27.1	22.56	23.82
24.03	26.72	25.1	28.03	29.38	23.05	24.17
23.07	26.44	24.53	26.82	27.15	22.02	23.86
24.24	27.44	26.26	27.83	27.6	23.7	24.52
23.96	25.79	24.24	28.7	29.05	22.07	24.27
23.38	26.21	24.63	27.95	29.63	22.01	24.03
22.69	24.85	24.1	26.47	27.21	21.39	23.24
23.79	27.87	25.47	27.1	27.57	23.16	24.54
23.78	25.73	24.58	27.41	29.47	23.15	23.97
23.81	26.51	25.34	27.32	28.05	22.98	25.35
23.95	26.81	25.19	27.1	27.09	23.01	23.8
25.91	28.72	29.68	29.79	30.31	24.78	26.78
23.84	26.36	24.6	26.99	27.38	22.62	24.14
23.38	26.63	24.8	26.79	28.18	22.35	23.61
23.3	25.82	24.03	27.65	28.79	22.18	23.85
24.87	27.98	26.95	28.85	28.39	23.59	24.97
23.13	25.87	24.91	26.89	27.9	21.98	23.47
23.22	27.37	24.97	26.31	26.73	22.94	23.82
23.7	26.05	25.55	27.99	29.62	22.65	23.54
24.05	27.42	25.22	27.34	29.62	22.66	23.78
24.1	26.32	25.68	28.33	30	23.25	23.44
23.71	26.69	25.75	27.42	28.23	22.9	23.75
23.39	26.51	24.56	28.33	30.1	22.27	24.36
23.51	25.41	24.29	28.53	29.77	22.02	24.17
23.69	25.84	24.75	27.47	29.15	22.62	23.74
23.53	25.84	24.6	27.88	30.11	21.73	23.45
23.94	27.47	25.06	27.05	27.51	23.01	24.69
23.92	25.83	25.54	27.72	30.35	22.34	24.13
23.5	25.3	24.26	27.57	29.87	22.34	24.13

TUG1.Hs002:	UCKL1.Hs002	ZFHX3.Hs001	ZNF839.Hs00217876_m1
26.17	26.65	25.66	28.53
26.57	26.81	25.64	28.99
26.11	26.43	25.3	28.24
26.74	27.3	25.58	29
26.61	26.32	25.11	28.09
27.16	27.18	26.61	29.11
26.77	26.95	25.6	28.76
26.49	26.2	25.25	28.6
25.81	25.96	24.45	27.49
26.88	27.13	25.71	28.31
26.92	26.37	25.1	28.5
27.07	26.96	25.5	28.55
26.5	26.68	25.94	28.38
28.94	29.3	28.42	30.31
26.74	26.55	24.98	28.5
25.92	26.98	25.18	27.97
26.5	26.32	24.75	27.89
27.25	28.55	27.01	29.99
26.11	26.41	25.07	27.96
26.34	26.82	25.43	28.19
26.44	27.32	25.8	28.04
26.8	27.17	25.74	28.7
26.53	27.54	25.75	28.45
26.47	27.62	26.28	28.94
27.03	27	24.65	28.69
26.97	26.27	25.52	27.8
26.16	26.96	25.19	28.32
26.2	26.62	25.2	27.95
26.76	26.94	25.75	28.53
26.28	26.99	25.59	28.9
26.36	26.47	25.31	28.46
