# Supplementary material for: Heterogeneity of Synovial Molecular Patterns in Patients with Arthritis
Source: PLoS One. 2015 Apr 30;10(4):e0122104. doi: 10.1371/journal.pone.0122104 (PMC4415786; doi:10.1371/journal.pone.0122104)
Supplement: S3 Table — (PDF) [file pone.0122104.s004.pdf]

High-density array probe sets and gene symbols displayed in Figure 2

Probe Sets	Gene Symbols
1405_i_at	CCL5
1552329_at	RBBP6
1554153_a_a	PHF21A
1555154_a_a	QKI
1555268_a_a	GRID1
1559814_at	---
1565705_x_a	---
1569472_s_a	TTC3
200680_x_at	HMGB1
200757_s_at	CALU
200795_at	SPARCL1
200972_at	TSPAN3
201503_at	G3BP1
201621_at	NBL1
201906_s_at	CTDSPL
202363_at	SPOCK1
202411_at	IFI27
203413_at	NELL2
203794_at	CDC42BPA
204258_at	CHD1
204415_at	IFI6
204529_s_at	TOX
204852_s_at	PTPN7
204891_s_at	LCK
205049_s_at	CD79A
205483_s_at	ISG15
205552_s_at	OAS1
205590_at	RASGRP1
206236_at	GPR4
207216_at	TNFSF8
207277_at	CD209
208475_at	FRMD4A
208677_s_at	BSG
208712_at	CCND1
208804_s_at	SRSF6
208819_at	RAB8A
209379_s_at	FAM190B
209406_at	BAG2
209537_at	EXTL2
209652_s_at	PGF
209932_s_at	DUT
210140_at	CST7
211275_s_at	GYG1

211710_x_at	RPL4
212394_at	KIAA0090
212615_at	CHD9
212725_s_at	TUG1
213193_x_at	TRBC1
214701_s_at	FN1
215992_s_at	RAPGEF2
216306_x_at	PTBP1
216920_s_at	TARP
217719_at	EIF3L
217795_s_at	TMEM43
217815_at	SUPT16H
217817_at	ARPC4
217945_at	BTBD1
218353_at	RGS5
218533_s_at	UCKL1
218543_s_at	PARP12
221709_s_at	ZNF839
221830_at	RAP2A
222440_s_at	THRAP3
222667_s_at	ASH1L
222728_s_at	TAF1D
222895_s_at	BCL11B
223667_at	FKBP7
225363_at	PTEN
225764_at	ETV6
225922_at	FNIP2
226218_at	IL7R
226409_at	TBC1D20
226702_at	CMPK2
226845_s_at	MYEOV2
226910_at	COMMD2
227030_at	IKZF3
227088_at	PDE5A
227224_at	RALGPS32
227677_at	JAK3
227894_at	WDR90
227908_at	TBC1D24
228760_at	SRSF8
229120_s_at	CDC42SE1
229450_at	IFIT3
229629_at	---
235355_at	CSRNP3
235419_at	---
236293_at	RHOH
236325_at	KIAA1377

236816_at	NAA25
240159_at	SLC15A2
241613_at	---
241905_at	PIK3C2A
242500_at	---
242625_at	RSAD2
242738_s_at	ZFHX3
244872_at	RBBP4
33132_at	CPSF1
37831_at	SIPA1L3
40837_at	TLE2
